# Synthetic enhancer design by *in silico* compensatory evolution reveals flexibility and constraint in *cis*-regulation

**DOI:** 10.1186/s12918-017-0485-2

**Published:** 2017-11-29

**Authors:** Kenneth A. Barr, Carlos Martinez, Jennifer R. Moran, Ah-Ram Kim, Alexandre F. Ramos, John Reinitz

**Affiliations:** 1 Committee on Genetics, Genomics, and Systems Biology, University of Chicago, Zoology 111, 1101 E 57th St, Chicago, 60637 Illinois USA; 2 Department of Ecology and Evolution, The University of Chicago, Chicago, 60637 Illinois USA; 3 Department Biochemistry and Molecular Genetics, Northwestern University, Chicago, 60611 Illinois USA; 4 Department Human Genetics, The University of Chicago, Chicago, 60637 Illinois USA; 5 Institute for Genomics & Systems Biology, The University of Chicago, Chicago, 60637 Illinois USA; 6 School of Life Science, Handong Global University, Pohang, 37554 Gyeongbuk South Korea; 7 Departamento de Radiologia – Faculdade de Medicina, Universidade de São Paulo & Instituto do Câncer do Estado de São Paulo, São Paulo, SP CEP, 05403-911 Brazil; 8 Escola de Artes, Ciências e Humanidades & Núcleo de Estudos Interdisciplinares em Sistemas Complexos, Universidade de São Paulo, Av. Arlindo Béttio, São Paulo, 1000 CEP 03828-000 SP Brazil; 9 Department Statistics, The University of Chicago, 5747 S. Ellis Avenue Jones 312, Chicago, 60637 IL USA

**Keywords:** Gene regulatory models, *Even-skipped* regulation, *Cis*-regulatory logic, Transriptional control, Synthetic enhancers, *Bicoid*, *Hunchback*, *Zelda*, *Stat92E*, *Dicheate*

## Abstract

**Background:**

Models that incorporate specific chemical mechanisms have been successful in describing the activity of *Drosophila* developmental enhancers as a function of underlying transcription factor binding motifs. Despite this, the minimum set of mechanisms required to reconstruct an enhancer from its constituent parts is not known. Synthetic biology offers the potential to test the sufficiency of known mechanisms to describe the activity of enhancers, as well as to uncover constraints on the number, order, and spacing of motifs.

**Results:**

Using a functional model and *in silico* compensatory evolution, we generated putative synthetic *even-skipped* stripe 2 enhancers with varying degrees of similarity to the natural enhancer. These elements represent the evolutionary trajectories of the natural stripe 2 enhancer towards two synthetic enhancers designed *ab initio*. In the first trajectory, spatially regulated expression was maintained, even after more than a third of binding sites were lost. In the second, sequences with high similarity to the natural element did not drive expression, but a highly diverged sequence about half the length of the minimal stripe 2 enhancer drove ten times greater expression. Additionally, homotypic clusters of Zelda or Stat92E motifs, but not Bicoid, drove expression in developing embryos.

**Conclusions:**

Here, we present a functional model of gene regulation to test the degree to which the known transcription factors and their interactions explain the activity of the *Drosophila even-skipped* stripe 2 enhancer. Initial success in the first trajectory showed that the gene regulation model explains much of the function of the stripe 2 enhancer. Cases where expression deviated from prediction indicates that undescribed factors likely act to modulate expression. We also showed that activation driven Bicoid and Hunchback is highly sensitive to spatial organization of binding motifs. In contrast, Zelda and Stat92E drive expression from simple homotypic clusters, suggesting that activation driven by these factors is less constrained. Collectively, the 40 sequences generated in this work provides a powerful training set for building future models of gene regulation.

**Electronic supplementary material:**

The online version of this article (doi:10.1186/s12918-017-0485-2) contains supplementary material, which is available to authorized users.

## Background

Enhancers, also known as *cis*-regulatory modules (CRMs), are DNA segments that recruit sets of sequence-specific transcription factors (TFs) in order to control the spatiotemporal expression of genes. These elements are critical in controlling cell fate in development [1] and are under selection [2–4]. More recently, genetic variation within enhancers has been widely implicated in common human disease [5, 6]. Predicting the effects of this *cis*-regulatory variation on local gene expression remains a challenging task. Even in the best studied enhancers, there is evidence of unknown function [7, 8], and it is not yet possible to reconstruct these elements from their constituent parts [9, 10].

The enhancer which drives the second of seven transverse stripes of *even-skipped* (*eve*) in the developing *Drosophila* blastoderm is among the most studied enhancers in all of biology. A deletion of a 480 bp fragment located 1.1 kb upstream of the transcription start site leads to loss of this stripe [11], and it is the smallest known fragment sufficient to drive reporter expression in a stripe 2 pattern [12]. Footprinting, TF knockouts [13] and site-directed mutagenisis [14] of this minimal stripe 2 element (MSE2) have identified 4 TFs that act through 12 sites in order to direct the stripe 2 pattern. MSE2 is broadly activated in the blastoderm through the activators Bicoid (Bcd) and Hunchback (Hb) and forms a stripe through repression by the factors Giant (Gt) on the anterior and Kruppel (Kr) on the posterior [12–15]. Despite being subject to such detailed molecular dissection, there are unexplained features of this enhancer. For instance, deletions of sequences outside the 12 footprinted sites all led to changes in function and additional TFs are required to prevent aberrant expression driven by this enhancer [8].

Enhancers integrate the simultaneous, opposing effects of both activators and repressors in order to determine specific expression levels. Thus, predicting the output of enhancers given any level of input requires quantitative methods. To address this, confocal microscopy has been used to generate spatial and temporal atlases of protein [16, 17] and mRNA [18, 19] levels at single nucleus resolution during the first 4 h of *Drosophila* development. Using transgenesis of enhancers driving reporter expression, the precise input-output function of enhancers can be measured. Sequence-level models (SLMs) of gene regulation have been used to describe this function as an emergent property of underlying TF binding sites [20–27]. Such models predict binding using thermodynamics and incorporate known, context-dependent rules of TF function, such as repression through short-range quenching [28–30]. SLMs have identified additional binding sites, regulators and interactions that are important in the control of MSE2 [20, 25].

Experiments with enhancers across *Drosophila* species suggest that there is considerable flexibility in the architecture of stripe 2 enhancers. Sequences that have diverged over tens of millions of years still drive stripe 2, despite a lack of sequence conservation [26, 31–34]. This functional conservation in the absence of sequence conservation indicates that there are many ways to construct a stripe 2 enhancer. SLMs trained on enhancer-reporter data from *D. melanogaster* have successfully predicted the activity of stripe 2 enhancers (S2Es) from distant Drosophilids [25] and identified accessible evolutionary paths that conserve expression through compensatory evolution [26]. This suggests that the context-dependent rules incorporated into SLMs are sufficient to describe the flexibility of MSE2 *cis*-regulatory logic.

While SLMs have been able to successfully describe the activity, evolution and flexible architecture of evolved enhancers, such enhancers represent only a small proportion of the sequences that are predicted to drive stripe 2 [35]. Instead, selection may obfuscate many constraints on the order and arrangement of TF motifs that give rise to functional S2Es by removing nonfunctional motif configurations from natural populations. This is suggested by the fact that sequences that lie outside of known binding motifs are necessary for expression [7, 8], as well as by past failures to generate synthetic *Drosophila* enhancers using reconstituted binding sites [9, 10].

Synthetic enhancers offer a potential means to address the extent to which known regulatory mechanisms represent a complete description of the *cis*-regulatory function, and to uncover hidden constraints on *cis*-regulatory architecture. While SLMs can be used to generate thousands of sequences that are predicted to drive virtually any pattern along the *Drosophila* anterior-posterior (AP) axis [35], the apparent success or failure of such sequences to drive the expected expression pattern is uninterpretable in the presence of a large number of changes from naturally selected sequence. Because the large number of sequence changes will tend to conflate those changes that are neutral with those that are critical, what is needed is a method in which functional changes can be attributed to a single or small number of sequence changes. Furthermore, because the minimum requirement for constructing a functional regulatory element is unknown, such changes must be made from the starting point of functional naturally selected sequence.

In this work we introduce a novel approach to the design of synthetic enhancers. Using synthetic compensatory evolution, we generated two series of S2Es with decreasing similarity to MSE2. These series address the extent to which SLMs describe the *eve* stripe 2 regulatory function and provides informative data when results differ from predictions. In total, we tested the activity of 40 synthetic putative regulatory sequences using site-specific integration [36] in developing *Drosophila* embryos. We collected quantitative expression data from 8 synthetic enhancers. We found that an SLM was able to successfully balance the effects of activators and repressors in order to maintain a stripe even after over a third of binding motifs were lost. We showed that a synthetic sequence half the size of the previously minimal stripe 2 element is able to drive stripe2 at more than 10 times the levels driven by MSE2. We showed that homotypic arrays of the activators Zelda (Zld) and Stat92E (Dst) are able to drive expression, but activation driven by Bcd and Hb is sensitive to the spacing, affinity, and orientation of sites. Additionally, we found that motif content not only controls mean expression levels, but also variability in expression within single embryos.

## Results

### Design of synthetic enhancers with decreasing similarity to MSE2

**Fig. 1:**
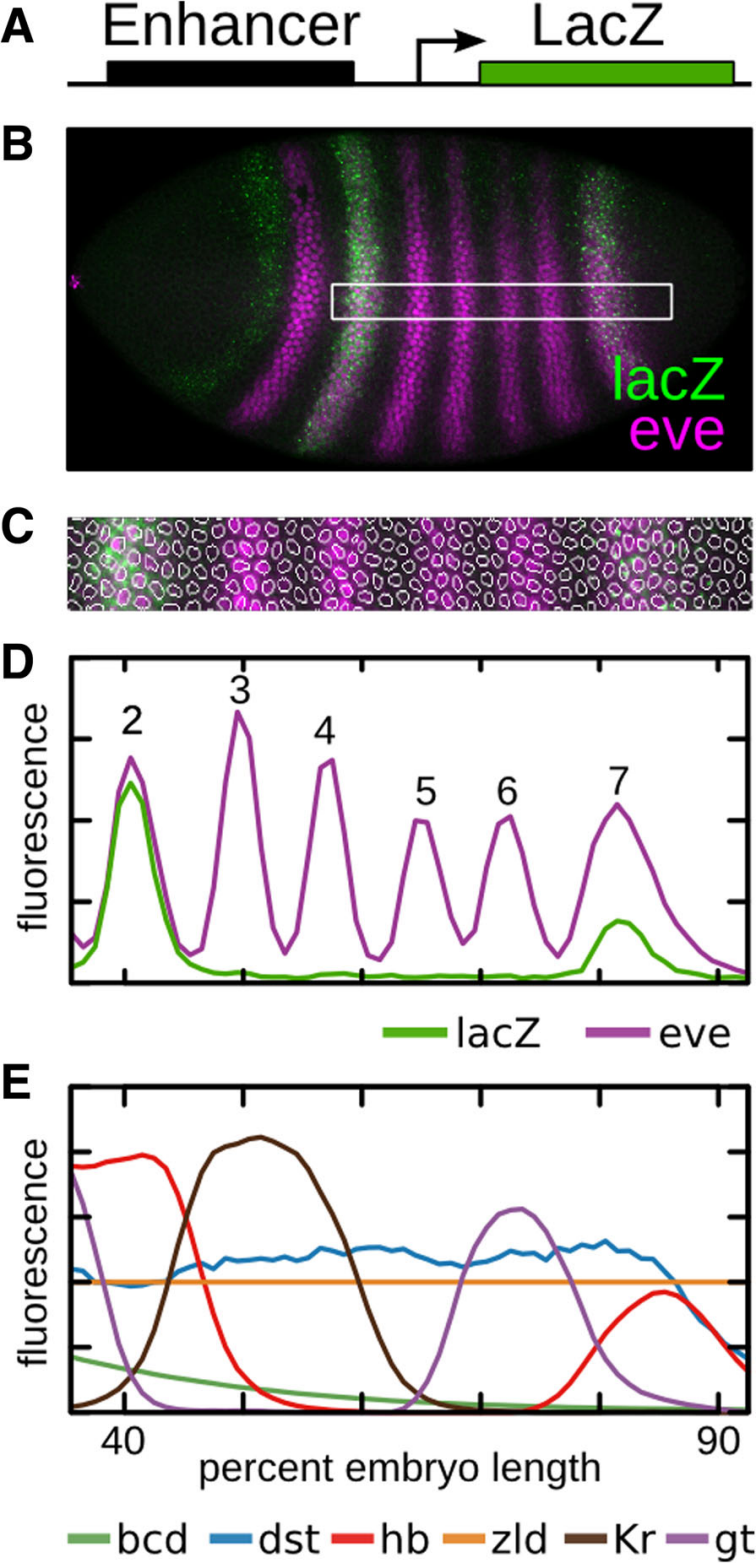
Enhancer quantification. **a** Regulatory sequences are cloned upstream of a *lac*Z reporter into the AttP2 site [36]. **b** Embryos are stained using fluorescent *in situ* hybridization (FISH) for *lac*Z and antibody staining for *eve* and imaged using confocal microscopy. **c** Nuclei are identified and levels of *lac*Z and *eve* are taken in a 10% DV stripe from 35.5% to 92.5% embryo length along the anterior-posterior axis at nuclear cycle 14, time class 6 [38]. **d** Multiple embryos are quantified to give an average expression level for any given enhancer along the AP axis. The identity of *eve* stripes 2 through 7 are indicated. **e** Previously quantified levels of transcriptional regulators are shown [16, 17, 25]

The rational design of synthetic enhancers requires a quantitative model that is able to balance the action of numerous activators and repressors acting on the same DNA sequence. The developing *Drosophila* embryo permits the input-output function of regulatory DNA to be assayed with single nucleus precision. In this work we placed test sequences upstream of a *lac*Z reporter using site specific integration in *Drosophila* embryos so that integration site effects were fixed and output could be quantitatively compared across lines (Fig. 1 a). The embryos were subsequently imaged in nuclear cycle 14 (C14), timeclass 6 (T6) [16, 17] for nuclei, *lac*Z and Eve protein (Fig. 1 b). At this point in development seven stripes of *eve* expression are clearly defined, but cross-regulation from other pair-rule genes is absent [11, 37]. Next, nuclei were segmented (Fig. 1 c) and data from multiple embryos were averaged to yield an expression profile for each enhancer in a 10% wide stripe along the AP axis (Fig. 1 d) [38]. We considered nuclei from 35.5 to 92.5% embryo length, where there is a clean functional distinction between the AP and dorsal-ventral axis. This profile was then registered to an atlas of protein levels [39]. The resulting dataset is a cell by cell assay of transcription under the control of quantified TFs, which can be used to obtain the input-output function of any DNA sequence (Fig. 1 e).

**Fig. 2:**
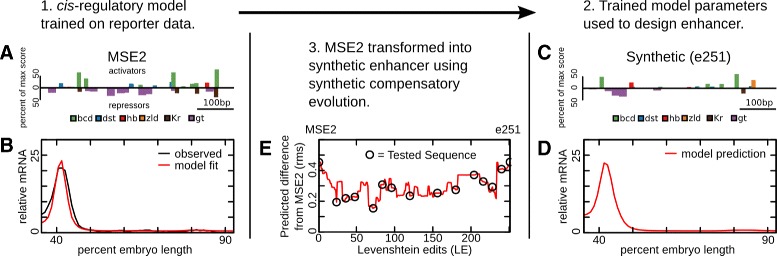
Design of a synthetic compensatory path. In order to generate a set of enhancers with decreasing similarity to MSE2, first a sequence level model of gene regulation (SLM) is trained. This model predicts expression from the underlying structure of binding sites. **a** The binding structure of the minimal stripe 2 element is shown. The height of bars represents the percent of the maximum log-likelihood score of a motif at each position. Putative activators are plotted on the positive axis and putative repressors on the negative axis. A subset of motifs are shown. Binding sites for all factors considered in this work are included in Additional file 1: Figures S5-S15. **b** Expression levels of enhancers are used to train an SLM. The observed expression levels (black) and model fit (red) are shown along the AP axis. **c** The binding structure of a putative synthetic stripe 2 element designed *in silico* using the SLM in panels A and A is shown, plotted as in panel A. **d** The predicted expression of the synthetic enhancer in C is shown plotted as in panel B. **e** A synthetic compensatory path is found that will transform MSE2 into the synthetic element while preserving predicted expression of a stripe. The root mean squared (rms) differences between predicted expression and MSE2 expression is shown as a function of number of sequence mutations. Each step along the *x*-axis represents a single nucleotide change to the previous sequence. A set of these elements (open circles) are selected for validation in vivo

In previous work, we generated quantitative models that explain the *cis*-regulatory function of eve stripe 2 from multiple species. In these works the kinetic parameters of an SLM were trained to the input output function of multiple enhancers (Fig. 2 a-b). In one instance, fusions of the *Drosophila even-skipped* stripe 2 and stripe 3 enhancers gave rise to novel expression patterns [40] that proved a rigorous training set for SLMs. An SLM trained on this data was able to predict expression pattern driven by S2Es from distant Drosphilid and Sepsid flies [25]. In another example, a model trained on S2Es from multiple Drosophilids identified putative ancestral S2Es and accessible evolutionary paths between them [26].

The parameters of SLMs generated in these studies provided a starting point for the design of synthetic regulatory sequences. Keeping the kinetic parameters of the SLMs fixed, we optimized DNA sequence using simulated annealing. We selected sequences that minimized the sum of squared differences between the expression of MSE2 and the predicted expression from one to seven models, each with its own set of kinetic parameters (see Fig. 2 c-d and “Methods” section). This process yielded sequences that were designed *ab initio* to drive expression in the pattern of *eve* stripe 2. Two such *ab initio* enhancers were generated and tested in vivo in the present study.

In order to generate a set of sequences with decreasing similarity to MSE2, we also generated sets of compensatory mutations that mutate MSE2 into each of the *ab initio* synthetic enhancers while maintaining stripe 2 expression. This was done by finding the single nucleotide changes required to mutate one sequence into the other, known as the Levenshtein edits (LE), and permuting their order such that predicted stripe 2 expression was conserved as much as possible at each edit [26, 35]. We call this set of edits a “neutral path.” Each neutral path used the same set of kinetic parameters as were used to design the *ab initio* synthetic construct at the end of the path. We selected paths of 15 and 11 putative enhancers respectively to test in vivo (Fig. 2 e).

The two *ab initio* synthetic enhancers generated in this work are separated from MSE2 by 251 and 272 LE respectively, and we call them e251 and s272. Sequence e251 was designed to drive expression from well separated binding sites using the *in silico* strategy described above. In contrast, sequence s272 was designed to be a “sub minimal” stripe 2 element that was as short as possible subject to the constraint that all TFs known to regulate stripe 2 could bind and exert their regulatory effects by mechanisms known to operate in S2E. This was done by arranging consensus bindings motifs for known regulators of stripe 2 by hand and then adjusting their affinity such that differences between stripe 2 expression and the model output of this enhancer were minimized. We discuss each of these sequences and the neutral paths in turn.

### Expression along the e251 synthetic compensatory path

**Fig. 3:**
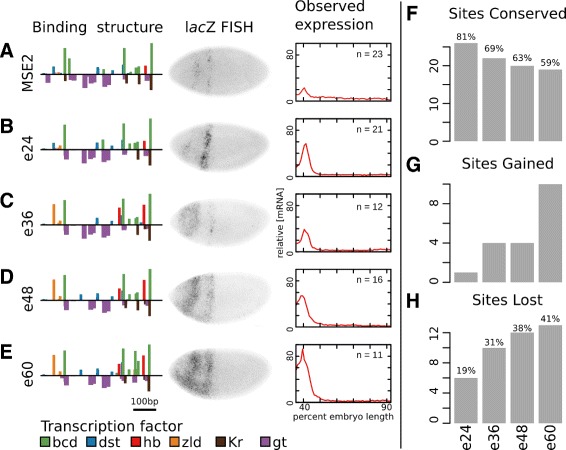
Expression along a synthetic compensatory path to e251. **a-e** For each sequence, the binding structure structure is shown (left). Height of bars is proportional to LLR of binding for each motif. A subset of motifs are shown. Binding sites for all factors considered in this work are included in Additional file 1: Figures S5-S15. We show FISH for *lac*Z driven by each of the sequences (center). We also show the quantitative level of mRNA driven by each enhancer along a 10% DV stripe from 35.5% to 92.5% embryo length (right). Data represents an average of *n* images, where the value of *n* is indicated. **a** MSE2. **b** e24 (24 LE removed from MSE2). **c** e36 (36 LE removed from MSE2). **e** e48 (48 LE removed from MSE2). **e** e60 (60 LE removed from MSE2). **f** The number of Bcd, Hb, Kr, and Gt binding sites in MSE2 that are maintained in each of the specified synthetic enhancers. The percent of binding sites (LLR>0) that are maintained is given above each bar. **g** The number of Bcd, Hb, Kr, and Gt binding sites (LLR>0) that are gained with respect to MSE2 are shown for each synthetic enhancer. **h** The number Bcd, Hb, Kr, and Gt binding sites (LLR>0) that are lost with respect to MSE2 are shown for each factor. blackThe percent of sites lost is given above each bar

The first four sequences tested along the neutral path to e251—at 24 LE (e24), 36 LE (e36), 48 LE (e48), and 60 LE (e60) from MSE2—all successfully balanced activation and repression in order to maintain expression of a stripe at 40% embryo length (Fig. 3). As predicted, each of these four sequences expressed at levels greater than MSE2 (Fig. 3 a-e). The remaining 11 sequences at greater than 72 LE did not drive expression in the modeled region. In addition to this region, many embroys also drove expression within the anterior portion of the embryo (Additional file 1: Figure S1A-E). The vector used in this work has previously been reported to drive an ectopic stripe anterior to *eve* stripe 1 [40]. To confirm that expression is driven by the vector, we generated a control construct that contained no enhancer. Embryos with this construct drove expression of an ectopic stripe (Additional file 1: Figure S2), albeit at levels considerably lower levels than in some tested synthetic enhancers.

To confirm that the sequence changes resulted in binding site turnover, we examined the gain and loss of binding motifs for the key regulators Bcd, Hb, Kr, and Gt. We identified a total of 32 binding motifs for these regulators at a log-likelihood ratio (LLR) [41] greater than zero. By 60 LE, 19 (59%) sites were maintained (Fig. 3 f), 10 sites were gained (Fig. 3 g), and 13 (41%) were lost (Fig. 3 g). This level of binding site turnover is comparable to that seen between *D. mel* and *D. erecta* (Additional file 1: Figure S3), which are several million years diverged [42] and show quantitative differences in expression driven by their respective S2Es [26].

### Known motifs cannot explain loss of expression after 60 LE

**Fig. 4:**
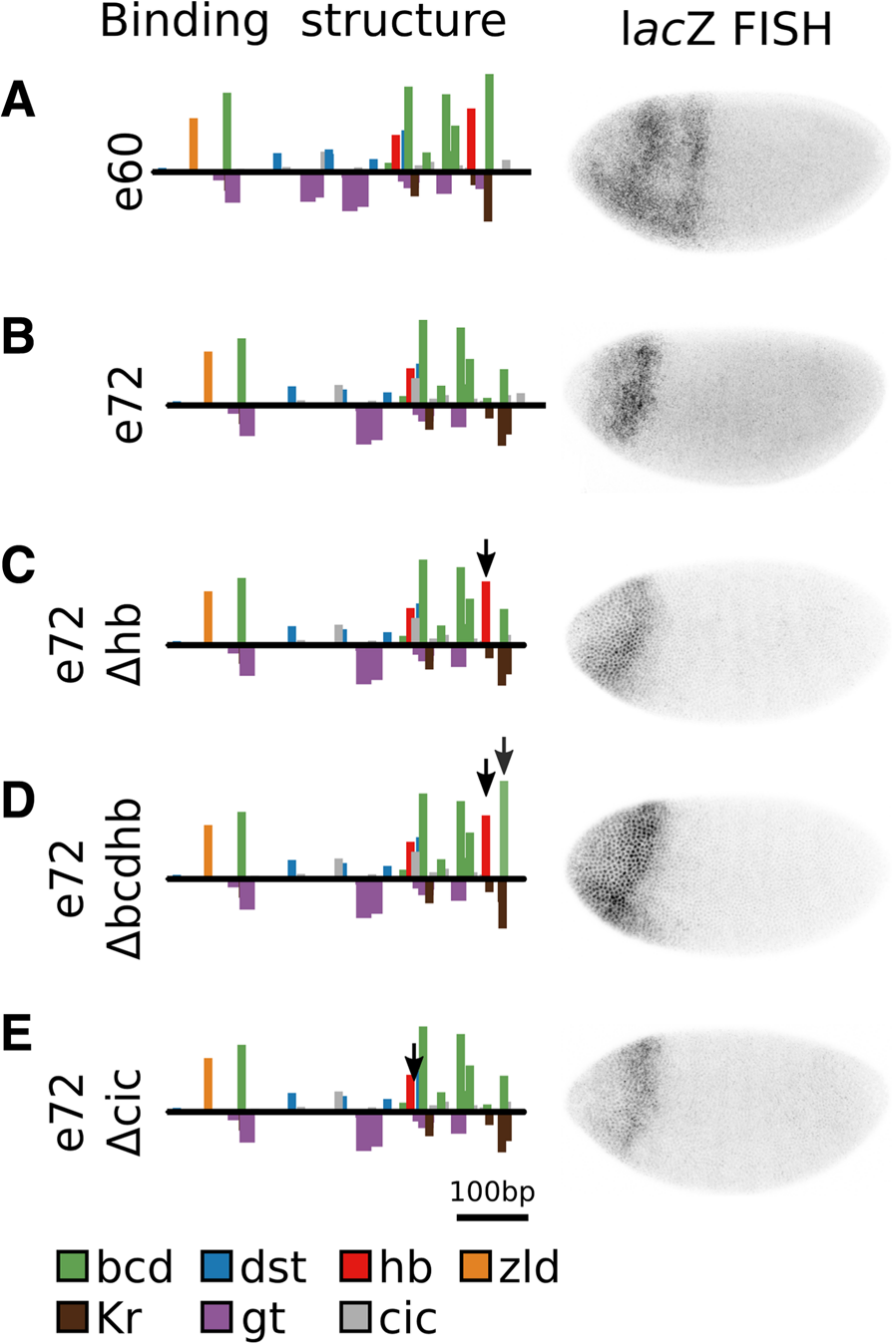
Known motifs cannot explain loss of expression after 60 LE. For each sequence, the binding structure structure is shown (left). Height of bars is proportional to LLR of binding for each motif. A subset of motifs are shown. Binding sites for all factors considered in this work are included in Additional file 1: Figures S5-S15. We also show FISH for *lac*Z driven by each of the sequences (right). **a** e60. **b** e72. This sequence is only 12 bp different than e60. **c** e72 with the affinity for a Hb site (arrow) restored to levels in e60. The construct does not drive expression. **d** e72 with the affinity for a Hb site and Bcd motif (arrows) restored to levels in e60. The construct does not drive expression. **e** e72 with the a Cic motif (arrow) removed, as in e60. The construct does not drive expression

While sequences at 60 LE or less drove stripe 2 expression, sequences at 72 LE or more failed to drive expression. We sought to identify which of the 12 sequence mutations were responsible for this change. Most model parameters predicted a reduction in expression at 72 LE (Additional file 1: Figure S1) as a result of the loss of a Hb motif and reduced affinity for Bcd. Recent work has highlighted the importance of the lost Hb site [25], making it a prime candidate for the change which caused loss of expression. The loss of the Hb motif was a result of a single nucleotide A>T change (ATAAAAA to ATATAAA). We reversed this change in e72 to restore the Hb motif. This did not rescue expression in e72 (Fig. 4 c).

A single nucleotide T>G change from e60 to e72 (GGATTA to GGATGA) disrupted a consensus Bcd motif. Hb, which is typically a repressor, is able to activate when bound near Bcd [25, 40, 43]. To test whether the loss of Bcd and Hb was responsible for loss of expression at e72, we restored both the Bcd and Hb sites in e72. The resulting sequence did not rescue expression driven by e72 (Fig. 4 d).

The remaining 10 nucleotide differences between e60 and e72 do not lead to appreciable differences in the predicted affinity for modeled TFs. We checked for predicted changes in binding preferences for factors within the Fly Factor Survey [44] that are maternally or ubiquitously expressed in the Berkeley Drosophila Genome Project [45]. A single candidate emerged. A single nucleotide T>A change (TGATTG to TGAATG) led to the creation of a site for the repressor Capicua (Cic) that is expressed throughout the entire modeled region. This repressor is reported to set borders of Bcd target genes [46], and its binding motif is present in all tested sequences at 72 or greater LE from MSE2. Removal of this site did not restore expression in e72 (Fig. 4 e).

### Expression along the s272 synthetic compensatory path

**Fig. 5:**
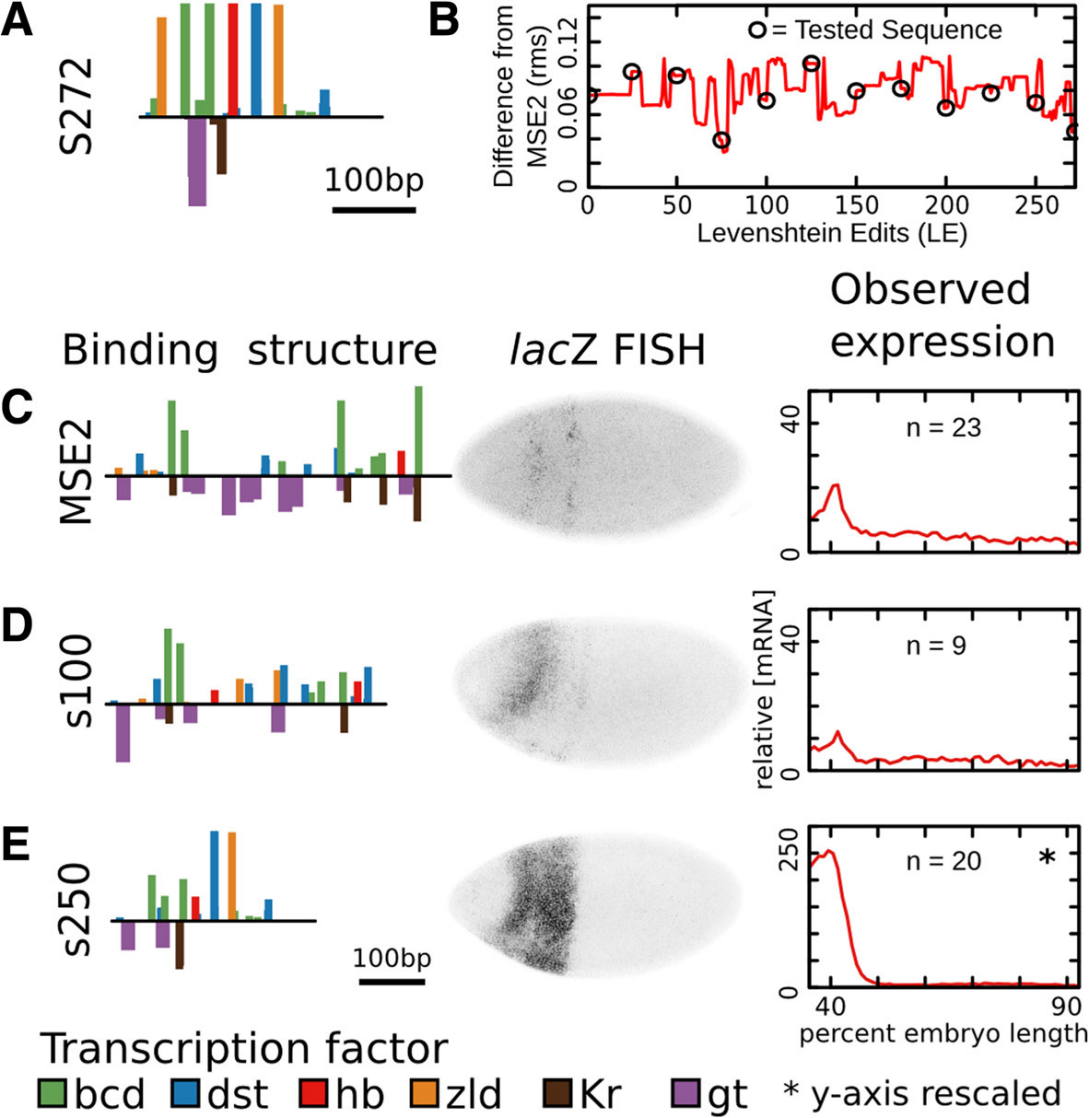
A 319bp synthetic enhancer drives stripe 2 at levels more than 10 times greater than MSE2. **a** The binding structure of a sequence designed, with model feedback, to drive expression of a stripe 2 pattern is shown. Height of bars is proportional to the LLR. Most sites represent consensus motifs for each factor. Binding sites for all factors considered in this work are included in Additional file 1: Figures S5-S15. **b** The predicted root mean squared differences from MSE2 expression along a series of 272 edits that would transform MSE2 into the sequence in panel A. 10 sequences (open circles) that were tested in vivo are shown. **c** The binding structure of MSE2 (left). FISH for *lac*Z driven by MSE2 (center). Quantitative levels driven by MSE2 (right). The number of averaged images, *n*, is indicated. **d** The binding structure of s100 (left). FISH for *lac*Z driven by s100 (center). Quantitative levels (right) driven s100, a sequence 100 edits from MSE2. This sequence drives expression of levels slightly less than MSE2. The number of averaged images, *n*, is indicated. **e** The binding structure of s250 (left). FISH for *lac*Z driven by s250 (center). Quantitative levels (right) driven by a s250, a 319 bp sequence 250 edits from MSE2. This sequence drives expression at levels more than 10 times greater than MSE2. The number of averaged images, *n*, is indicated

The sequence s272 was designed to contain the motifs and interactions currently known to be essential for stripe 2 expression (Fig. 5 a). The sequence contains a single motif for Dst, which is known to be essential for expression of eve stripe 3 [47] and is a major activator of zygotic expression [48]. It contains two adjacent motifs for the activator Bcd, which binds to DNA cooperatively [49]. It contains a single Hb site, which activates expression of eve stripe 2 when bound near Bcd [20, 25]. These four activator motifs are flanked by two Zld motifs, which has been reported to open chromatin [50, 51]. Finally, the sequence contains motifs for the repressors Gt and Kr, which set the boundaries of stripe 2 expression [12–15, 20]. In addition to this sequence, we designed and tested the activity of ten sequences in a set of 272 LE that mutate MSE2 into this synthetic enhancer while conserving stripe expression (Fig. 5 b, Additional file 1: Figure S4 i).

While the designed enhancer did not drive reporter expression (Additional file 1: Figure S4), two tested sequences drove expression of a stripe at 40% embryo length. A sequence at 100 LE (s100) drove weak expression of stripe 2, despite having lost 22 of 32 (69%) of binding motifs for the factors Bcd, Hb, Gt, and Kr (Fig. 5 d). Another sequence, 250 LE from MSE2 (s250), drove very strong expression of a stripe at 40% embryo length despite having only 2 (6%) motifs conserved with respect to MSE2. At 319 bp in length, with the majority of motifs falling in a less than 200 bp cluster, this sequence is significantly smaller than the 480 bp minimal stripe 2 element, yet drove expression at levels more than 10 times greater than MSE2 (Fig. 5 e).

### Homotypic clusters of Zelda and Stat92E drive embryonic expression

**Fig. 6:**
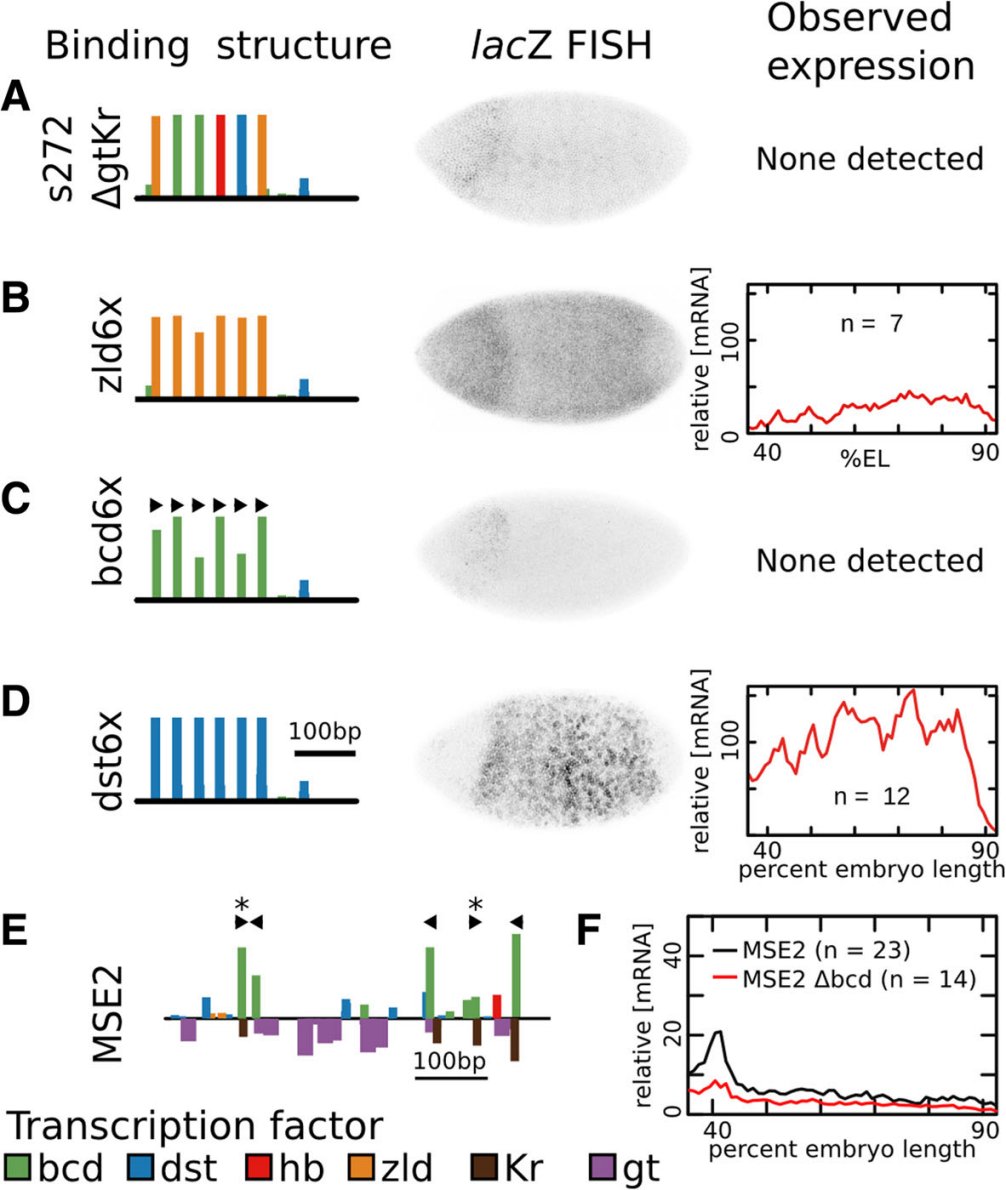
Homotypic clusters of Zld and Dst, but not Bcd drive embryonic expression. **a-d** For each sequence, the binding structure structure is shown (left). Height of bars is proportional to LLR of binding for each motif. A subset of motifs are shown. Binding sites for all factors considered in this work are included in Additional file 1: Figures S5-S15. We show FISH for *lac*Z driven by each of the sequences (center). We also show the quantitative level of mRNA driven by each enhancer along a 10% DV stripe from 35.5% to 92.5% embryo length (right). Data represents an average of *n* images, where the value of *n* is indicated. **a** s272 with sites for repressors Gt and Kr removed. **b** Each motif in s272 *Δ*gtKr was replaced with a motif for Zld, preserving inter-motif sequences. The resulting enhancer drives expression across the entire length of the embryo. **c**. Each motif in s272 *Δ*gtKr was replaced with a motif for Bcd, preserving inter-motif sequences. Arrow represent the orientation of binding motifs. The resulting sequence did not drive expression. **d** Each motif in s272 *Δ*gtKr was replaced with a motif for Dst, preserving inter-motif sequences. The resulting enhancer drives expression across the middle of the embryo. The number of averaged images, n, is indicated. **e** The binding structure of MSE2 is shown with arrows indicating the orientation of Bcd motifs in the sequence. **f** The quantitative expression driven by MSE2 and MSE2 with the motif orientations indicated in panel E reversed. The resulting sequence has the same predicted affinity for Bcd, but drives less expression than MSE2

The sequence s272 did not drive expression despite being separated by only 22 LE from a strong enhancer. We hypothesized that loss of expression could be due to an imbalance between activation and repression. To test this hypothesis, we generated a variant of the designed enhancer that eliminated motifs for Kr and Gt (s272 *Δ* *gt* *Δ*Kr). The resulting sequence failed to drive expression (Fig. 6 a).

In order to determine which TFs are capable of driving expression alone, we generated sequences with homotypic clusters of 6 motifs for Zld, Bcd, and Dst. Starting from the previous construct, we replaced each of the 6 motifs with a motif for the specified factor, keeping any intermotif sequences constant. Some small differences from consensus motifs were necessary to prevent the creation of repressor motifs. Homotypic clusters of Zld drove moderate expression (Fig. 6 b) and homotypic clusters of Dst drove strong levels of expression (Fig. 6 d).

### Bcd binding orientation is important for MSE2 function

While homotypic clusters Zld and Dst drove reporter expression in developing embryos, 6 Bcd motifs failed to drive expression (Fig. 6 c). The fact that Bcd immunoprecipitation preferentially pulls down sequences with Bcd in the head to head orientation [52] suggests that Bcd orientation may be an important factor. This point is strengthened by the fact that sequences with different Bcd orientation and spacing drove different levels of expression in fly embryos [49, 53]. In order to test the role of Bcd orientation in MSE2, we reversed the orientation of two Bcd sites, keeping the affinities of all sites constant (Fig. 6 e). The resulting sequence drove significantly lower levels of expression than MSE2 (Fig. 6 f).

**Fig. 7:**
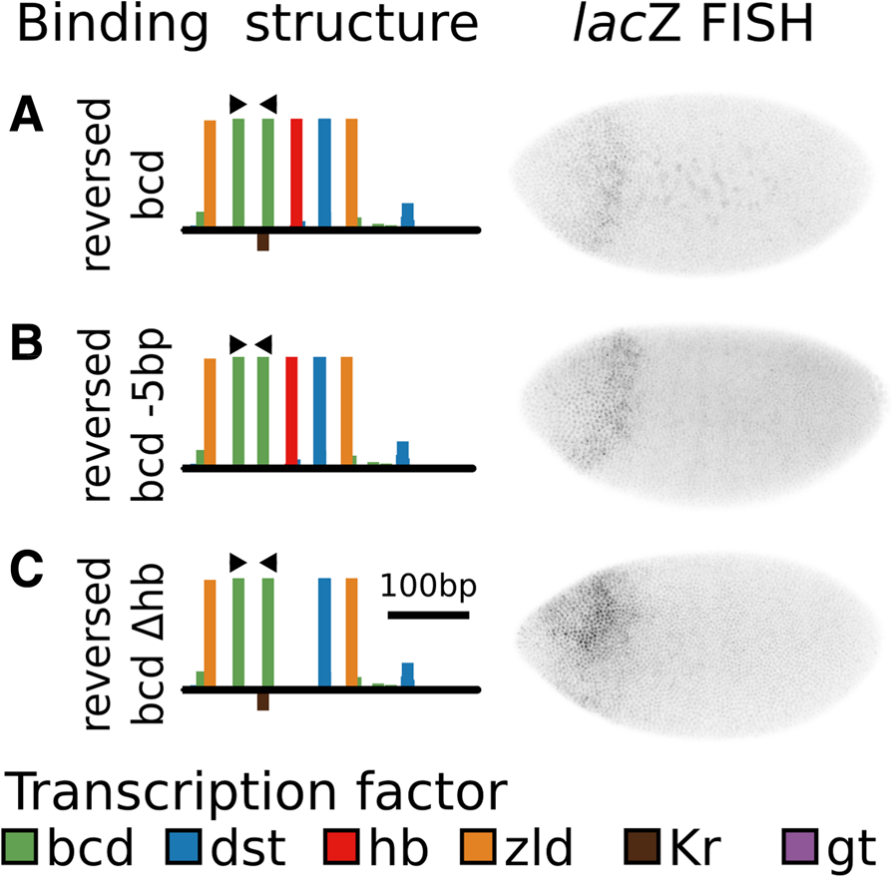
Bcd orientation and spacing does not rescue s272 *Δ* *gt* *Δ*Kr. For each sequence, the binding structure structure is shown (left). Height of bars is proportional to LLR of binding for each motif. A subset of motifs are shown. Binding sites for all factors considered in this work are included in Additional file 1: Figures S5-S15. We also show FISH for *lac*Z driven by each of the sequences (right). **a** s272 *Δ* *gt* *Δ*Kr with the second Bcd motif orientation reversed. The resulting enhancer does does not drive expression. **b** s272 *Δ* *gt* *Δ*Kr with the 5 bp of inter-motif spacer removed. The resulting enhancer does not drive expression. **c** s272 *Δ* *gt* *Δ*Kr with the second Bcd motif orientation reversed and Hb site deleted. The resulting enhancer does not drive expression

In order to test the role of Bcd orientation in s272 *Δ* *gt* *Δ*Kr, we generated a sequence that reversed the orientation of a Bcd motif (Fig. 7 a). This sequence did not restore expression. In order to test whether helical orientation on DNA prevented these sites from binding cooperatively, we removed 5bp of inter-motif DNA. This sequence did not restore expression (Fig. 7 b). Finally, to test whether Hb was not being coactivated, and thus preventing expression through quenching, we tested a sequence that reversed a Bcd motif orientation and removed the Hb motif. This sequence did not restore expression (Fig. 7 c).

### Bicoid, Huncback, and Dicheate are essential for expression driven by s250

While various orientations of Bcd and Hb did not restore expression in s272, the sequence s250 drove strong expression despite being only 22 LE different in sequence. The fact that Bcd binding orientation is important in MSE2 suggested that the specific orientation of Bcd and Hb motifs in s250 is essential. Additionally, a strong binding motif for the TF Dicheate (Dic) was lost between s250 and s272.

**Fig. 8:**
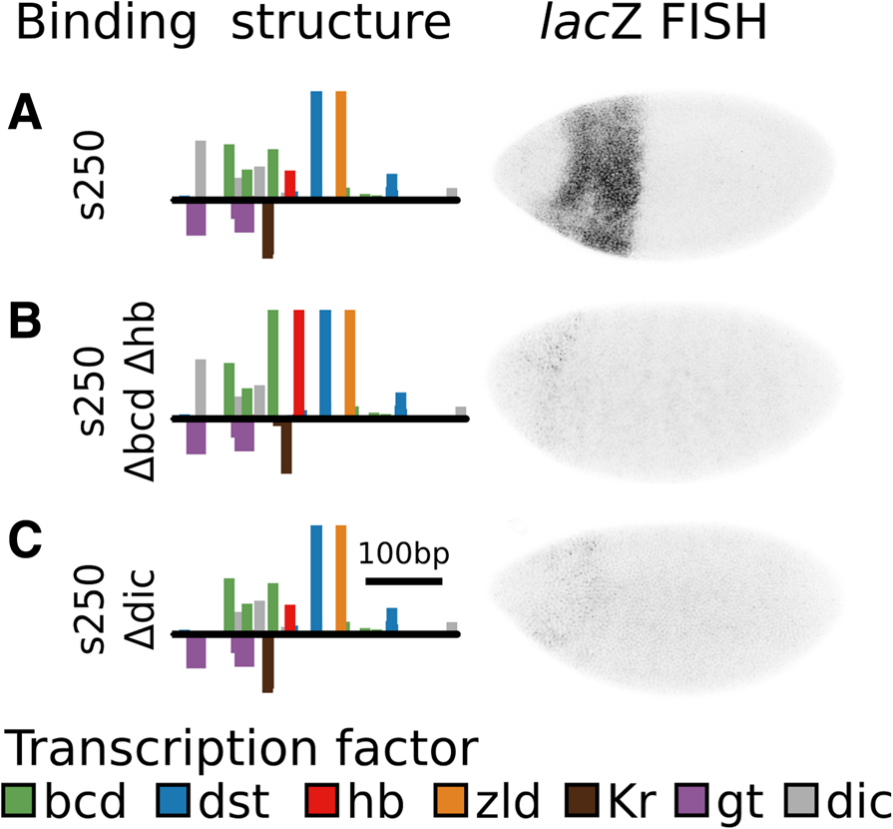
Bicoid, Huncback, and Dicheate are essential for expression driven by s250. For each sequence, the binding structure structure is shown (left). Height of bars is proportional to LLR of binding for each motif. A subset of motifs are shown. Binding sites for all factors considered in this work are included in Additional file 1: Figures S5-S15. We also show FISH for *lac*Z driven by each of the sequences (right). **a** s250. The the factor Dicheate has been included in the binding structure in grey. The enhancer drives strong expression. **b** s250 with Bcd and Hb orientation reverted to the orientation present in s272. The resulting enhancer does not drive expression. **c** s250 with the a single Dicheate site removed. The resulting enhancer does not drive expression

We tested whether each of these changes was responsible for loss of expression individually. Editing a single Bcd and single Hb site and their inter-motif sequence led to a complete loss of expression driven by the sequence (Fig. 8 b). Surprisingly, a change of only 3bp that removes a single motif for Dicheate also led to complete loss of expression (Fig. 8 c).

### Motif content controls variability in expression within embryos

**Fig. 9:**
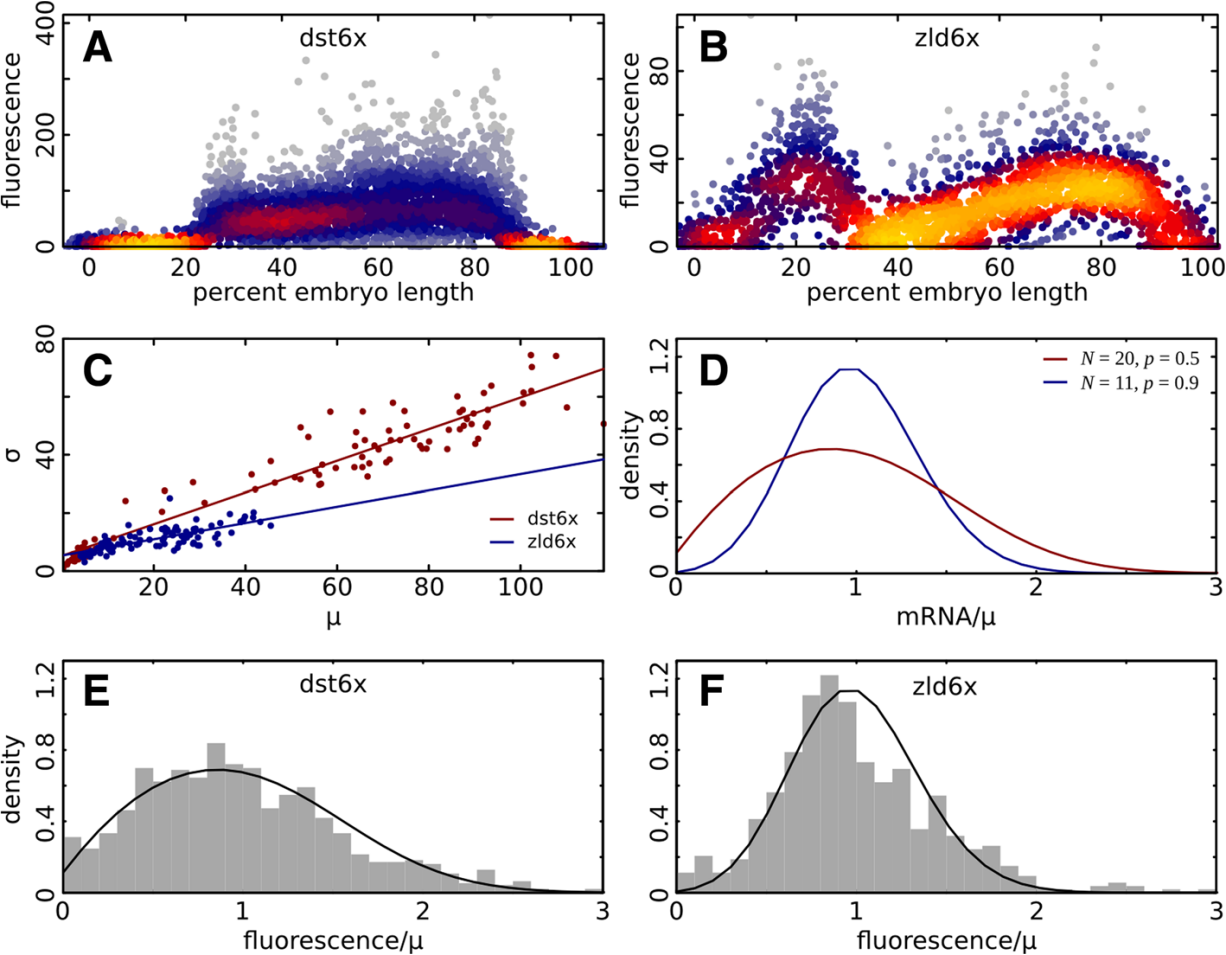
Motif structure controls variability in expression **a** Individual nucleus fluorescence levels driven by the construct dst6x. Expression data on each embryo was scaled to minimize the the sum of squared pairwise differences in fluorescence intensity between embryos in 1% bins along the AP axis. Points are colored according to their local density. **b** Expression levels driven by the construct zld6x, plotted as in panel A. **c** To test whether differences in standard deviation can be explained by different mean expression levels, the standard deviation as a function of the mean expression is plotted for each 1% bin along the AP axis for both dst6x (red) and zld6x (blue). The relationship between standard deviation and mean is linear with different slopes for each construct. **d** Two distributions of mRNA count, divided by the mean, in a stochastic transcription model in which the number of transcripts and the ON-OFF state of the promoter are coupled random variables (see Methods). The parameter *N* represents the strength of transcription when the gene is in the ON state, and *b* is the probability of finding the gene in the ON state. **e** Fluorescence values of nuclei were divided by the mean levels in each 1% bin from 60 to 80% embryo length. The resulting distribution in fluorescence about the mean for dst6x is shown. The distribution with *N*=20 and *p*=0.5 from panel D is also shown (black line). **e** The distribution of fluorescence about the mean for zld6x is shown. There is a significant difference in the variance of the zld6x and dst6x distributions (Fligner-Killeen test, *p*=1.7×10^−8^). Additionally, the distribution with *N*=11 and *p*=0.9 from D is shown (black line)

We noted a difference in the visual appearance of mRNA fluorescence *in situ* hybridization (FISH) for expression driven by homotypic clusters of Dst compared to Zld (Fig. 6 b, d), which seemed to indicate a high level of variation in expression between adjacent nuclei in the construct driven by Dst. In order to investigate within embryo variation we first adjusted for differences in mean expression between embryos (“Methods” section), and then considered the expression in individual nuclei across the AP axis when driven by homotypic clusters of Dst (Fig. 9 a) and Zld (Fig. 9 b). The levels of expression driven by Dst appeared to have both a higher mean and greater variability than expression driven by Zld. To test whether the higher mean levels could explain variability, we tested the relationship between the mean and standard deviation in each line. We found that there was a linear relationship between the mean expression and standard deviation of expression in 1% bins along the AP axis, but the slope of this line for the Dst driven enhancer was nearly double that of the Zld driven enhancer (0.28 to 0.54) (Fig. 9 c), indicating that greater expression variability cannot be explained by difference in the mean.

In order to observe the shape of the distribution independent of the mean, we divided the fluorescence values by the mean levels at each AP 1% bin from 60 to 80% embryo length. The resulting distribution in fluorescence about the mean is wider when driven by Dst than when driven by Zld (Fig. 9 e-f).

## Discussion

Synthetic biology affords the opportunity to rigorously test constraints on the number, order, and types of TF binding sites required to drive specific spatial and temporal expression of genes. In this work we generated 40 synthetic sequences using a model of gene regulation that captures known chemical mechanisms and rules that govern the architecture of enhancers. These enhancers, which had varying degrees of similarity to MSE2, were constructed in order to address the degree to which these mechanisms and rules are sufficient to describe the activity of enhancers. We found that while the model successfully predicted the activity of several enhancers, incongruities point to new molecular players and mechanisms that are required to predict regulatory function.

The mechanisms included in our model are DNA binding, steric competition for DNA binding, cooperative binding, short-range repression, direct repression, and coactivation of Hb by Bcd or Caudal. This set has been sufficient to explain the flexibility evident in enhancer sequence divergence over the course of evolution [25, 26]. The fact that S2E expression was maintained in the first tested synthetic compensatory path, even after 41% of binding sites for key regulators were lost, suggests that these mechanisms explain much, but not all, of the function of S2E.

### Additional factors

Despite this initial success, only the first 4 of 15 tested synthetic sequences in the path to e251 successfully drove expression in nuclear cycle 14. e60 drove expression and e72 failed to drive expression. We analyzed the 12 nucleotide changes that led to loss of expression in e72. Of these 12 changes, 3 were in known binding motifs. Restoring these three changes did not restore expression. This result indicated that there are either unknown motifs which have been gained or lost, or that the edits resulted in other structural changes to DNA that disrupted function.

The result that additional factors regulate MSE2 has been suggested by other work. Andrioli et al. [8] showed that five deletions outside of 12 footprinted sites for Bcd, Gt, Kr and Hb all disrupted the function of MSE2. Similarly, Vincent et al. [9] showed that an enhancer reconstituted with all 12 footprinted sites failed to drive expression. In addition to these 12 footprinted motifs, this work also modeled non-footprinted sites as well as sites the factors Zld, Dic, Dst, Knirps, and Tailless. Despite considering considerably more putative regulators of MSE2 than these previous works, this list of regulators is likely still incomplete. This is also the case for other other *Drosophila* enhancers, where function was found to reside within most inter-motif sequences [7, 10]. Alternatively, DNA features such as GC content and dinucleotide content may affect reporter activity through structural effects or by modulating affinity for nucleosomes [54].

### Constraints on enhancer architecture

Every enhancer in the neutral paths considered in this work was constructed to contain a similar balance of bound activators and repressors. Despite this fact, these sequences drove vastly different levels of expression in developing embryos. This suggests that there are additional interactions between bound transcription factors that modulates their activity. We characterized one particular interaction in this work, Bcd cooperativity, that has a constraint not considered in the model. We found that changing the orientation of two Bcd sites within MSE2 disrupted activity of the enhancer, leading us to conclude that pairwise cooperative binding of Bcd requires a pair of sites with opposite orientation on the same strand.

We also discovered a molecularly uncharacterized component of interactions between Bcd and Hb. In sequences containing only six binding sites for known activators, we were unable to find combinations and orientations of Bcd and Hb that drove expression in developing embryos, even in the absence of known repressors (Fig. 7), and despite the sequence being compatible with expression when the six motifs were substituted with either Zld or Dst. Despite this, changing 15 nucleotides between s250 and s250 *Δ* *bcd* *Δ*hb in such a way as to increase the affinity and spacing of a Bcd and Hb site was able to render the strongest enhancer assayed in this work non-functional (Fig. 8b). Collectively, this suggests that spacing and orientation of Bcd and Hb sites is critical in controlling levels of expression. While there are many permissible configurations, as evidenced by binding turnover in evolution, there may be many more configurations that are not permissible. Further work with synthetic sequences that exhaustively tests regulatory output as a function of distance and orientation will be required to precisely define this interaction.

This conclusion that enhancers are highly sensitive to the arrangement of binding sites is supported by other work with synthetic enhancers. In *Drosophila* embryos, synthetic sequences containing homotypic and heterotypic clusters of binding sites were sensitive to small changes in intermotif distances and motif orientations [55]. Furthermore, these constraints were tissue dependent, suggesting that changing concentrations of cofactors may affect constraints on *cis*-regulatory architecture. Similarly, synthetic sequences designed to probe the distance dependency of quenching found that this function is not monotonic [30] and depended on orientation [56]. In mouse, analysis of synthetic sequences containing various complexities of motif structure identified numerous pairwise synergistic interactions [57]. Moreover, synthetic sequences containing eight motifs drove highly variable expression depending on the arrangement [57].

### Expression variability

We found that synthetic enhancers drove different levels of within-embryo expression variability that is independent of the mean (Fig. 9 c, e-f). These distributions are reminiscent of distributions seen in a stochastic transcription model in which the number of transcripts and the ON-OFF state of the promoter are coupled random variables [58, 59]. We found that the width of distributions of mRNA levels, scaled to the mean, is altered by varying the probability that a gene is actively transcribing, *p* (Fig. 9 d). While the mean expression level driven by six Dst motifs is far higher than that driven by six Zld motifs, the distribution driven by Zld is less variable than that driven by Dst. This indicates that the the probability that the promoter is in the ON state is lower when driven by Dst than by Zld. This difference could be explained by the role of Zld as a chromatin remodeler. While Dst can drive high levels of transcription, it must compete with nucleosomes for binding. Where Dst has displaced nucleosomes high levels of transcription are achieved, while adjacent nuclei are inactive. In contrast, if Zld can more easily displace nucleosomes, all nuclei will have consistent levels of Zld binding. This leads to a high probability of the gene being in the ON state, even though Zld activates transcription more weakly than Dst.

## Conclusions

Interpreting and predicting the function of regulatory DNA, directly from sequence, remains a fundamental challenges in molecular genetics. It will require understanding the ways in which bound transcription factors interact in order to modulate gene expression. Functional models of gene regulation incorporate known transcription factors and their interactions in order describe and predict the function of regulatory elements.

In this work we tested the ability of a functional model of gene regulation to predict the expression driven by putative synthetic enhancers that have varrying degrees of similarity to MSE2. Initial success indicated that these factor mechanisms explain much of the function of MSE2, however we found evidence for both new factors and interactions that have not been incorporated into previous models. Specifically, we show that orientation of Bcd sites is critical in MSE2 (Fig. 6 f), and that the interaction between Bcd and Hb is highly sensitive to spacing or affinity (Fig. 8 b). In contrast, simple homotypic clusters of Zld and Dst drove expression, indicating that these factors may be less constrained with respect to the spacing and orientation of motifs.

Typically, models of *Drosophila* gene regulation have been trained on functional enhancers, but it is important to consider both positive and negative data in training sets. The sequences generated in this work contain many instances in which active elements are separated from inactive elements by only a few nucleotides. This property will rigorously constrain future models of gene regulation.

## Methods

### Design of synthetic enhancer sequences

The method of optimizing sequence given a set of kinetic parameters is discussed in depth in Martinez et al. [35]. In brief, seven parameters sets, characterized in three previous works [25, 26, 35], were used to generate the sequences in this work. The parameters for these models are given in Additional file 1: Table S1. Parameter sets 1, 2, and 3 are described Kim et al. [25], where they are called model 01, 06, and 07 respectively. Parameter set 4 was trained using all the data from Kim et al. as well as the expanded model in Martinez et al. [26]. This fit used the PWMs for Hb and Bcd, that are reported in Martinez et al. Parameter sets 5 and 6 were trained to the same data used by Kim et al. but used a different PWM for Bcd [60]. Parameter set 7 was obtained by training with the PWMs and data used in Kim et al., with the addition of Zld as a uniformly expressed activator at a constant level of 100. The Zld and Cic PWMs are from the Fly Factor Survey [44]. All PWMs used in this study are given in Additional file 1.

The synthetic sequence e251 was designed using all seven parameter sets. In order to generate a sequence with well separated sites, we used the cost function

1 $$ E = \Big(\sum_{i} (x_{i} - y_{i})^{2} \Big) + \beta o,   $$

where *x*_*i*_ and *y*_*i*_ are the model output and data respectively, *β* is a configurable parameter, *o* is the number of overlapping motifs, and collectively *β* *o* is a penalty for overlapping binding motifs. We defined two motifs as overlapping if the end to end distance between their footprints was within 5 nucleotides, and we set *β* to be 1% of the maximum possible score, corresponding to *x*_*i*_=255 for all *x*. To generate e251, we minimized the mean cost function

2 $$ E_{\text{consensus}} = \frac{1}{7} \sum_{i=1}^{7}E_{i},  $$

where *i* denotes a parameter set from the set of seven described above. The initial sequence used was random.

The synthetic sequence s272 was designed by starting with a 258 bp DNA having the structure shown in Fig. 5 a, but with each binding site having a maximum affinity consensus sequence. This sequence drove a predicted stripe 2 pattern a few nuclei anterior of the observed pattern when assessed using parameter set 2. The affinities for repressors Gt and Kr were then reduced such that the pattern was predicted to drive expression of a stripe at the position of stripe 2. This was accomplished with a single nucleotide change to the Gt consensus motif (TTACGCAAT to TTACGCAA*A*) and three changes to the Kr consensus (TAACCTTTC to *A*AACCC*A*TT*T*).

### Design of enhancers by synthetic compensatory evolution

The method of generating synthetic compensatory paths is discussed in depth in two previous works [26, 35]. In brief, we select the synthetic sequence (e251 or s272), identify the number of single nucleotide edits required to mutate MSE2 into this sequence, then permute the order of edits such that at each step we minimize a cost function. We define the function

3 $$ F = \sum_{i} \left(\frac{x_{i}}{\max_{j} x_{j}}-\frac{y_{i}}{\max_{j} y_{j}}\right)^{2} \times \text{Penalty},  $$

where *x*_*i*_ is the predicted model output and *y*_*i*_ is the data. This function standardizes both data and output on a 0 to 1 scale. We penalize model predicted expression less than data with the multiplicative penalty,

4 $$ \text{Penalty} = \left\{\begin{array}{ll} \frac{\max_{i} y_{i}}{\max_{i} x_{i}} & \max_{i} x_{i} < \max_{i} y_{i}\\ 1 & \max_{i} x_{i} \geq \max_{i} y_{i} \end{array}\right. \,.  $$

For the path to s272, we minimize the the function

5 $$ F_{\mathrm{s272}} = \sum_{i=1}^{272}F_{i},  $$

where *i* denotes the sequence after *i* LE given the permutation being scored. Only model 2 was used in scoring.

For the path to e251, which uses consensus design, we minimized the function

6 $$ F_{\mathrm{consensus e251}} = \sum_{i=1}^{251} \sum_{j=1}^{7}F_{ij},  $$

where *i* denotes the sequence after *i* LE and *j* is a parameter set from the set of seven previously described.

### Generation of reporter constructs

Reporter constructs where generated using a p*CaSpeR* backbone (GeneBank X81644.1) containing the promoter and first 22 amino acids of *eve* fused to *lac*Z, generated by Small et al. [40]. An AttB sequence was inserted into the multiple cloning site using the restriction enzyme *Xba*1 for insertion in the AttP2 landing site on chromosome 3 [36]. The enhancer sequence was extended by PCR primers containing overlap with this vector (Additional file 1). The vector was then digested by enzymes *Eco*R1 and *Xho*1 and the enhancer was inserted using Gibson assembly [61]. The resulting vector was injected into flies of the genotype P{nos-phiC31\int.NLS}X, P{CaryP}attP2 by Rainbow Transgenics. Quantitative data was collected from these lines as previously described [38].

### Sequences used in this work

The sequences of all 40 enhancers generated in this work are included in Additional file 1. Additionally, expression data are provided in Additional file 2.

### Analysis of binding site conservation

In order to determine the number of binding sites gained, lost, or conserved between two sequences we first performed a pairwise alignment between two sequences using the R package Biostrings. The log-likelihood ratio (LLR) of binding was calculated at every position in each aligned sequence. Sequences were called binding sites if the LLR was greater than zero. In order to accommodate gaps in sequence alignments, sites were considered conserved if they aligned within 3 bp. Sites were considered lost if there was no site with LLR greater than 0 within 3 bp on the corresponding aligned sequence. For the background distribution we use the frequencies of nucleotides in the *Drosophila* genome (*P*_bg_(*A*)=*P*_bg_(*T*)=0.297, *P*_bg_(*C*)=*P*_bg_(*G*)=0.203).

### Scaling of data for variation analysis

Variation in expression can be due to effects both within and between embryos. In order to remove the between embryo effects, we introduced a scaling factor for each embryo which multiplies the fluorescence measurements across the entire AP axis. We then optimized the scaling factors for each embryo in order to minimize the sum of squared differences in fluorescence measurements between embryos of the same genotype. This was subject to the constraint that the sum of scaling factors equals the number of embryos of that genotype. The scaled data from multiple embryos was then pooled for subsequent analysis.

### Theoretical distribution of mRNA

The steady-state distribution of mRNA counts has been previously derived for a stochastic transcription model in which the number of transcripts and the ON-OFF state of the promoter are coupled random variables ([62], Eq. 29). This distribution is defined by three variables: *p* gives the probability of the promoter being in the ON state, *N* gives the transcription rate when the promoter is in the ON state, and *b* gives the rate of switching between ON and OFF states. Ramos et al. ([62], Eq. 29) gives the distribution of mRNA when the promoter is in the OFF state, *α*_*n*_, or ON state *β*_*n*_. Here we report the total distribution *ϕ*_*n*_=*α*_*n*_+*β*_*n*_, keeping the parameter *b* fixed at *b*=4. The mean number of mRNA is given by *μ*=*N* *p*.

## Additional files

Additional file 1 Supplementary Materials. A PDF containing supplementary figures, tables, position weight matrices used, and sequences generated in this work. (PDF 4800 kb)

Additional file 2 Quantified expression patterns. An xls file containing averaged fluorescence measurements of the expression pattern driven by the enhancer sequences used in this work. (XLS 664 kb)

## Abbreviations

Bcd: Bicoid
C14: Nuclear cycle 14
Cic: Capicua
CRM: *cis*-regulatory module
Dic: Dicheate
Dst: *Drosophila* Stat92E
*eve*: *even-skipped*
FISH: Florescence *in situ* hybridization
Gt: Giant
Hb: Hunchback
Kr: Kruppel
Kni: Knirps
LE: Levenshtein edits
LLR: Log-likelihood ratio
MSE2: The *even-skipped* minimal stripe element 2
rms: root mean squared
S2E: Stripe 2 element. Any natural or synthetic sequence that drives the *even-skipped* stripe 2 pattern
T6: Timeclass 6
TF: Transcription factor
Zld: Zelda
