# Supplementary material for: Synthetic enhancer design by in silico compensatory evolution reveals flexibility and constraint in cis-regulation
Source: BMC Syst Biol. 2017 Nov 29;11:116. doi: 10.1186/s12918-017-0485-2 (PMC5708098; doi:10.1186/s12918-017-0485-2)
Supplement: Supplementary file 1 — Supplementary Materials. A PDF containing supplementary figures, tables, position weight matrices used, and sequences generated in this work. (PDF 4800 kb) [file 12918_2017_485_MOESM1_ESM.pdf]

# Contents


**1 Supplementary Materials** **2**

1.1 Supplementary Figures and Tables . . . . . . . . . . . . . . . . . . . . . . . . 2

1.2 Position Weight Matrices Used . . . . . . . . . . . . . . . . . . . . . . . . . . 20

1.3 Sequences . . . . . . . . . . . . . . . . . . . . . . . . . . . . . . . . . . . . . 21


# List of Figures


S1 Figure S1. Expression and prediction on path to e251. . . . . . . . . . . . . 4

S2 Figure S2. mRNA FISH of a reporter containing no enhancer. . . . . . . . . 4

S3 Figure S3. Conservation of motifs in S2Es . . . . . . . . . . . . . . . . . . . 5

S4 Figure S4. Expression and prediction along a path to s272. . . . . . . . . . . 7

S5 Figure S5. Binding sites for Bcd . . . . . . . . . . . . . . . . . . . . . . . . 8

S6 Figure S6. Binding sites for Cad . . . . . . . . . . . . . . . . . . . . . . . . 9

S7 Figure S7. Binding sites for Cic . . . . . . . . . . . . . . . . . . . . . . . . 10

S8 Figure S8. Binding sites for Dic . . . . . . . . . . . . . . . . . . . . . . . . 11

S9 Figure S9. Binding sites for Dst . . . . . . . . . . . . . . . . . . . . . . . . 12

S10 Figure S10. Binding sites for Gt . . . . . . . . . . . . . . . . . . . . . . . 13

S11 Figure S11. Binding sites for Hb . . . . . . . . . . . . . . . . . . . . . . . 14

S12 Figure S12. Binding sites for Kni . . . . . . . . . . . . . . . . . . . . . . . 15

S13 Figure S13. Binding sites for Kr . . . . . . . . . . . . . . . . . . . . . . . 16

S14 Figure S14. Binding sites for Tll . . . . . . . . . . . . . . . . . . . . . . . 17

S15 Figure S15. Binding sites for Zld . . . . . . . . . . . . . . . . . . . . . . . 18


# List of Tables


S1 Table S1. Model Parameters. . . . . . . . . . . . . . . . . . . . . . . . . . . 20

# 1 Supplementary Materials

## 1.1 Supplementary Figures and Tables

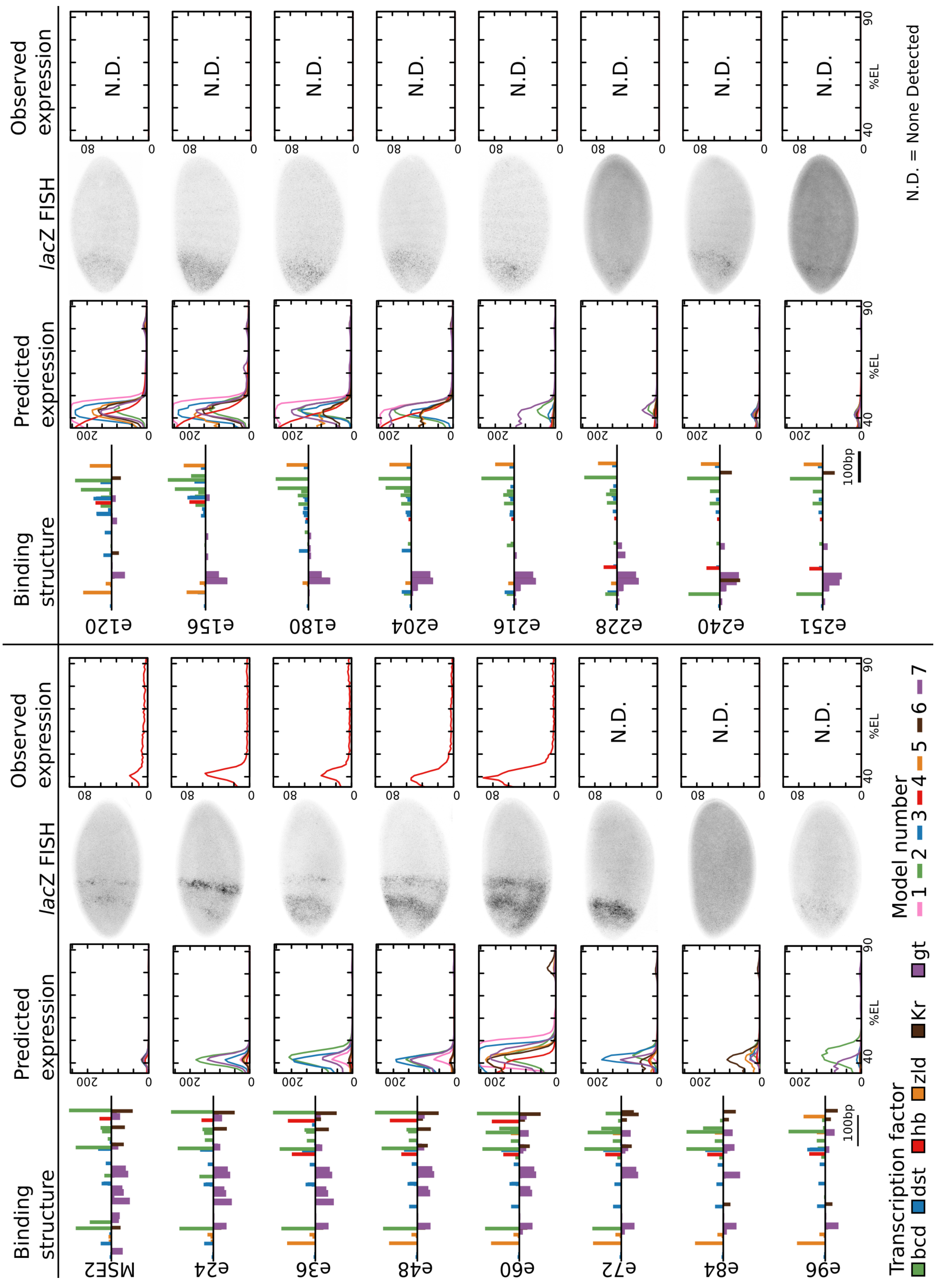


**Figure S1.** Continued on next page...

**Figure S1. Expression and prediction on path to e251.** Continued from previous page. For all tested enhancers the following are shown: (Column 1) Binding structure of tested enhancers. Activators are plotted on the positive $y$-axis and repressors on the negative. Bar height is proportional to the log-likelihood ratio of binding. (Column 2) The predicted output in each of 7 models used in the design of the sequences. (Column 3) The *lac*Z mRNA expression of a representative embryo. (Column 4) The quantitative level of mRNA expression for each line along a 10% DV stripe from 35.5% to 92.5% embryo length.

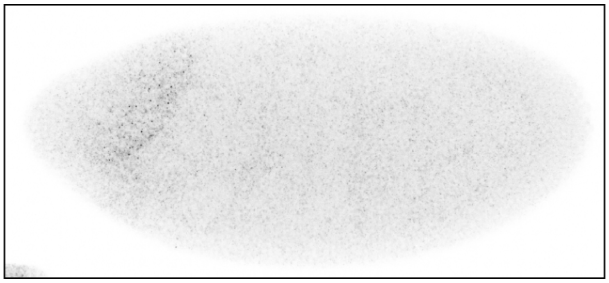

**Figure S2. mRNA FISH of a reporter containing no enhancer.** We generated a vector, as previously described, that contains no inserted putative enhancer and inserted the sequence into the Attp2 landing site. The resulting construct drives weak expression of an ectopic stripe anterior to *eve* stripe 1, confirming that this expression pattern is driven, at least in part, by sequences present on the vector.

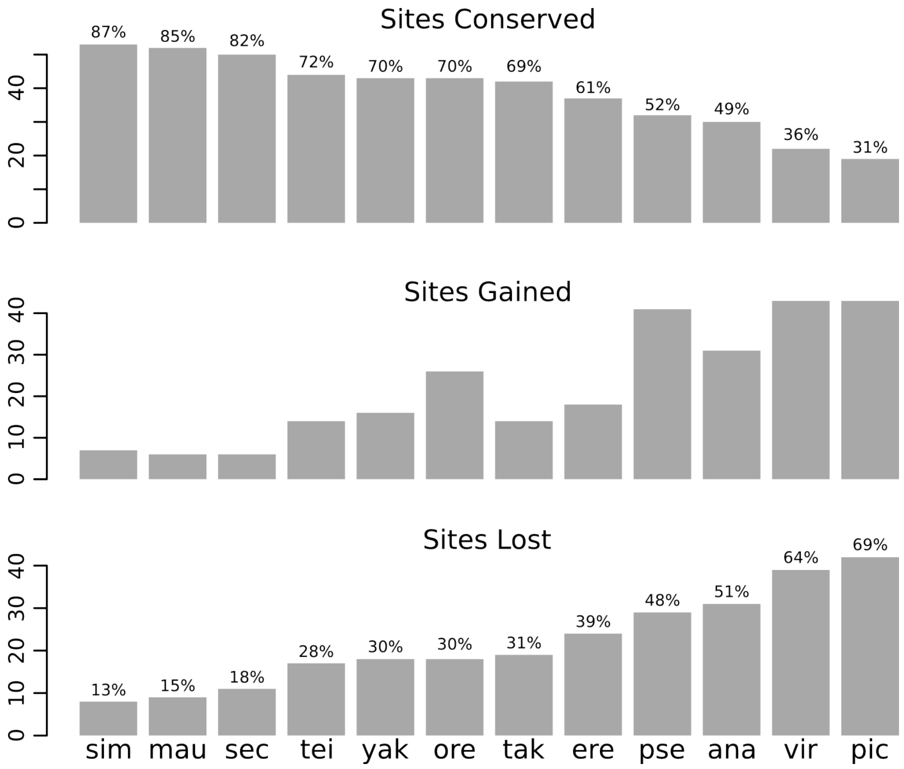


**Figure S3. Conservation of motifs in S2Es.** (Top) The number of binding motifs at LLR greater than 0 for the factors Bcd, hb, Kr, and gt that are conserved with S2E are shown for each putative S2E. The percent conservation is given above each bar. (Middle) The number of binding motifs (LLR>0) for the same 4 factors that are gained are shown for each putative S2E. (Bottom) The number of motifs (LLR>0) for the 4 factors that are lost are shown for each factor. The percent of sites lost is given above each bar. The sequences of each are reported in Kim et. al.[**?** ].

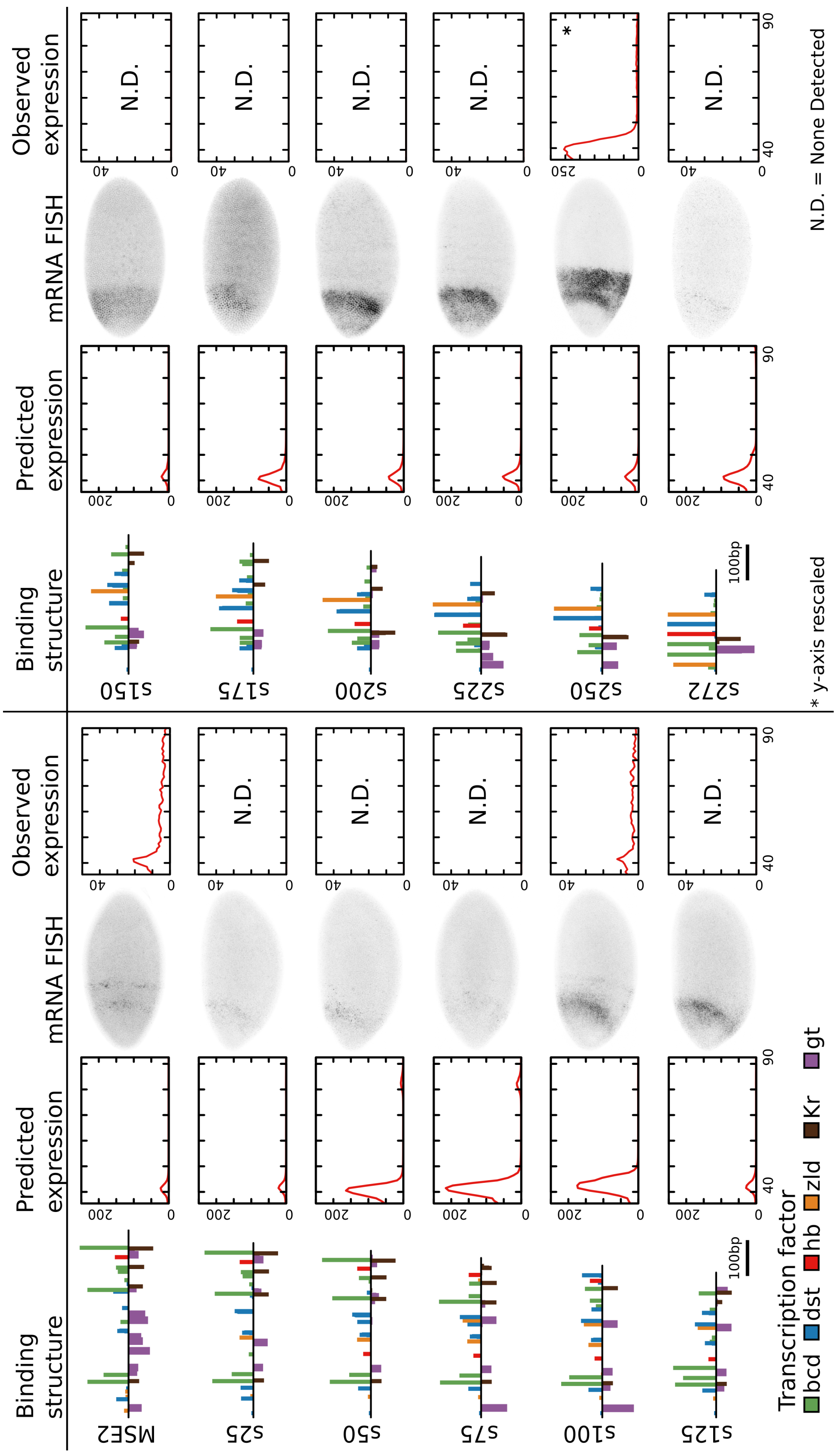


**Figure S4.** Continued on next page...

**Figure S4. Expression and prediction along a path to s272.** Continued from previous page. For all tested enhancers the following are shown: (Column 1) Binding structure of tested enhancers. Activators are plotted on the positive $y$-axis and repressors on the negative. Bar height is proportional to the log-likelihood ratio of binding. (Column 2) The predicted output in the model used in the design of the sequences. (Column 3) The *lac*Z mRNA expression of a represenative embryo. (Column 4) The quantitative level of mRNA expression for each line along a 10% DV stripe from 35.5% to 92.5% embryo length.

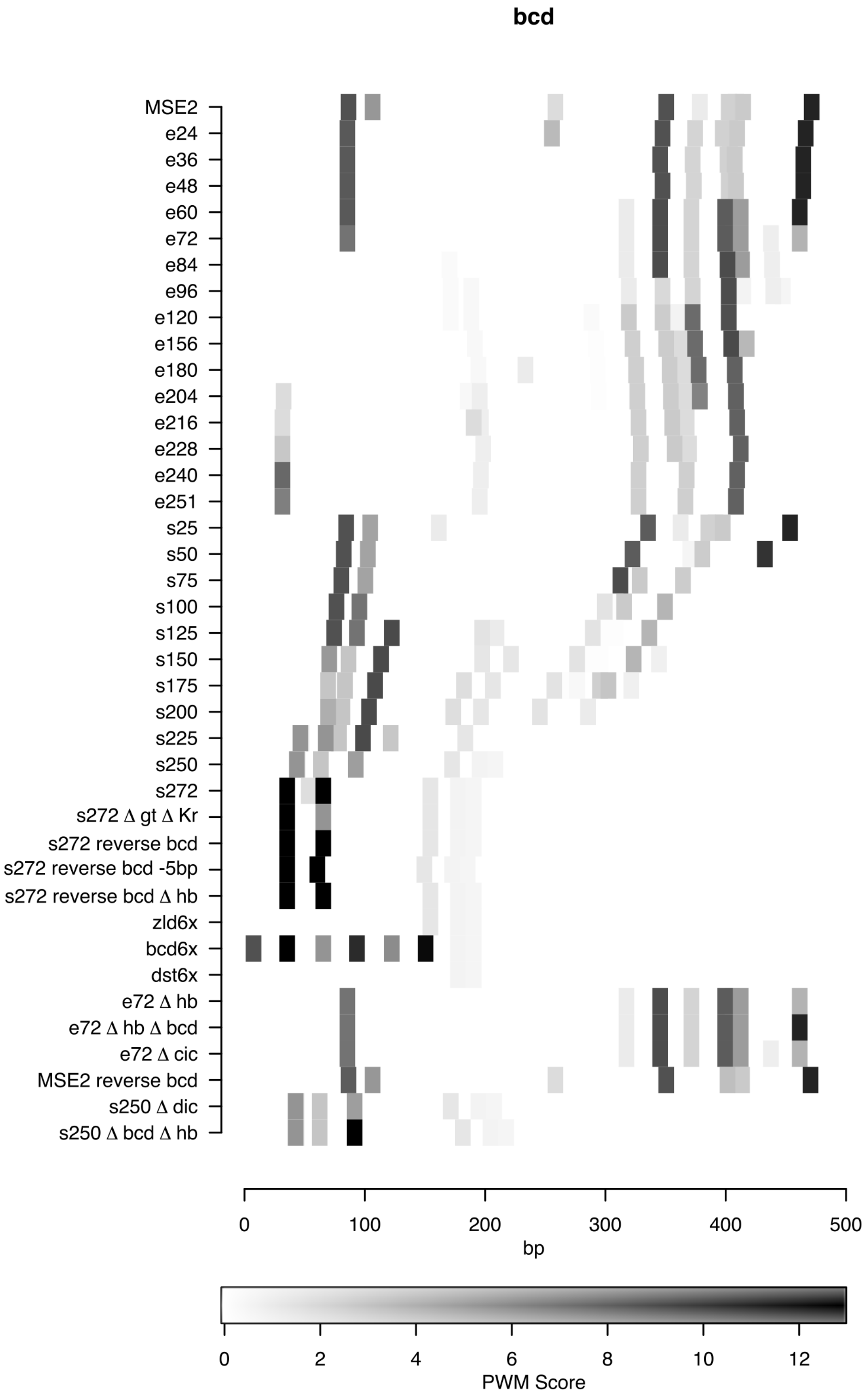


**Figure S5. Binding sites for Bcd** For each sequence used in this work, we calculated the Bcd PWM score at each position. Motif matches (score>0) are indicated by rectangles that are shaded according to the PWM score, with the legend under the $x$-axis.

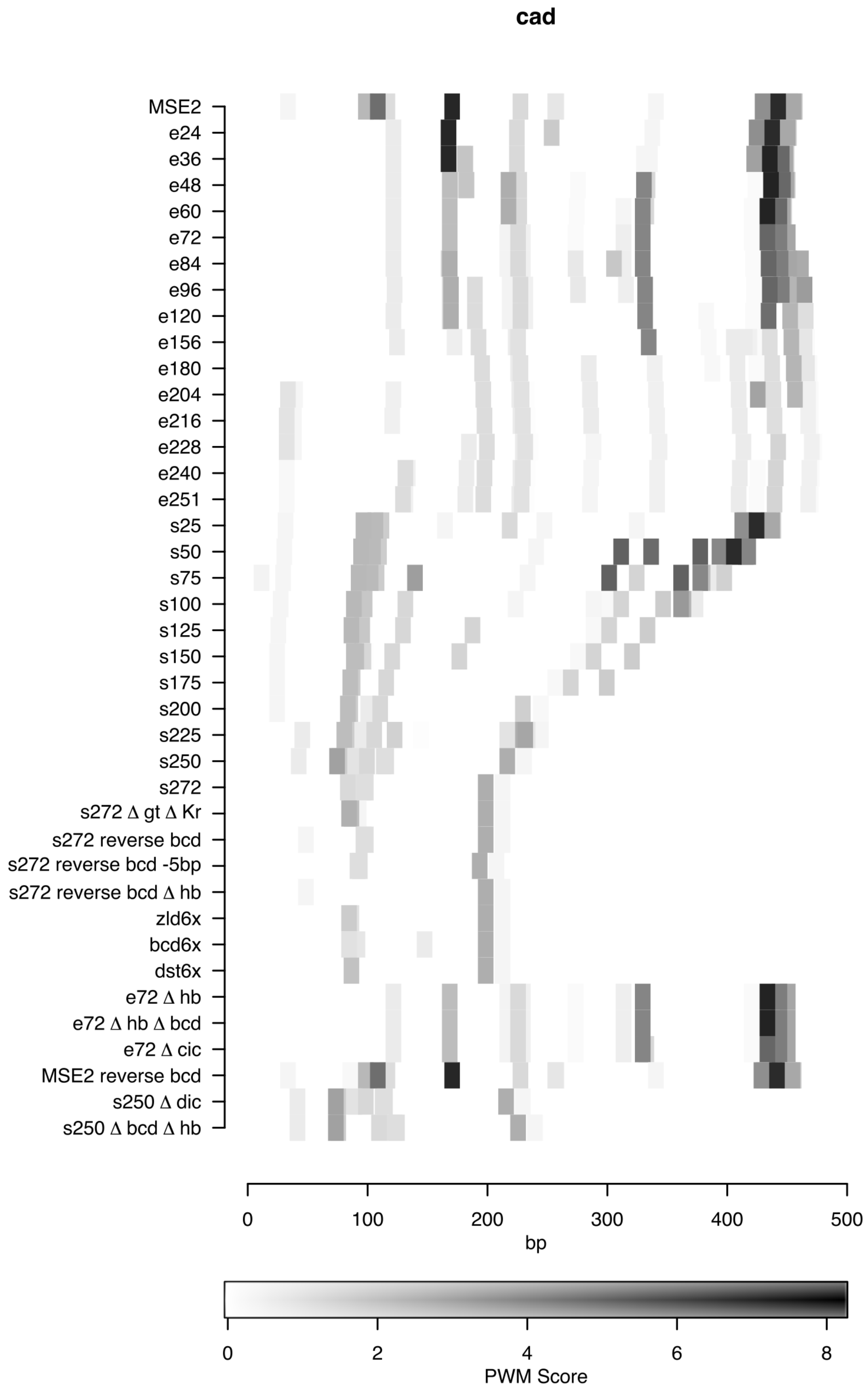


**Figure S6. Binding sites for Cad** For each sequence used in this work, we calculated the Cad PWM score at each position. Motif matches (score>0) are indicated by rectangles that are shaded according to the PWM score, with the legend under the $x$-axis.

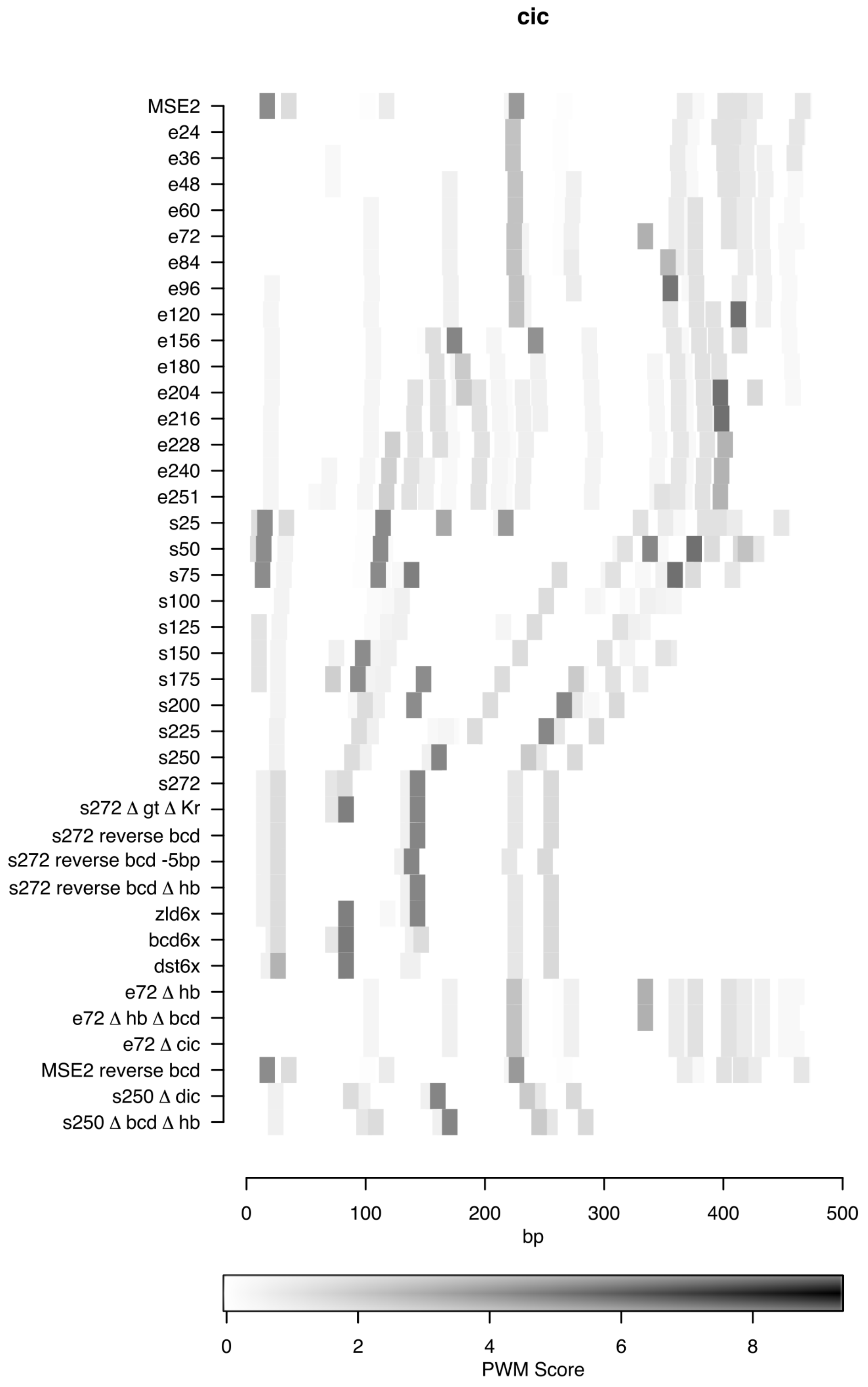


**Figure S7. Binding sites for Cic** For each sequence used in this work, we calculated the Cic PWM score at each position. Motif matches (score>0) are indicated by rectangles that are shaded according to the PWM score, with the legend under the $x$-axis.

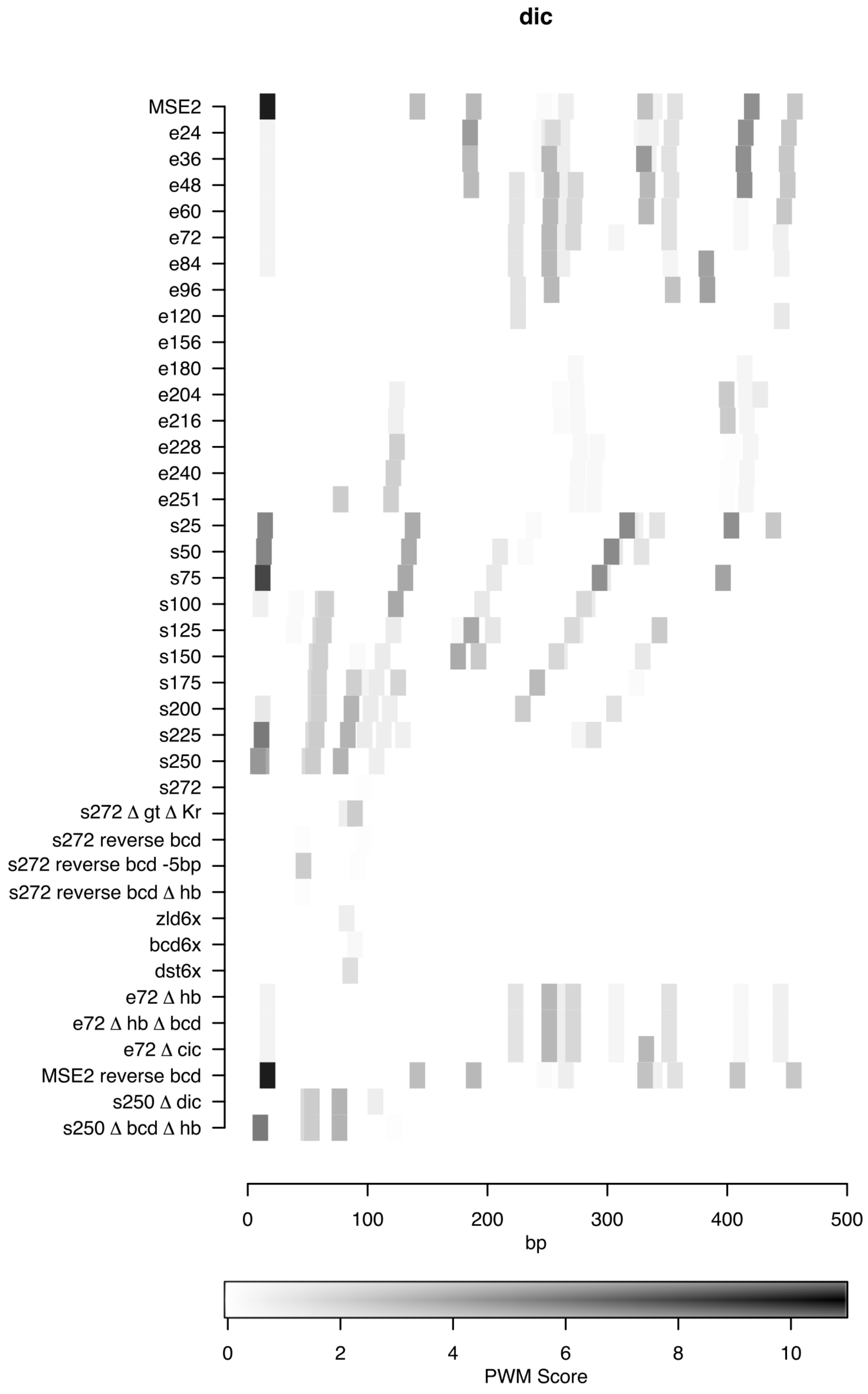


**Figure S8. Binding sites for Dic** For each sequence used in this work, we calculated the Dic PWM score at each position. Motif matches (score>0) are indicated by rectangles that are shaded according to the PWM score, with the legend under the $x$-axis.

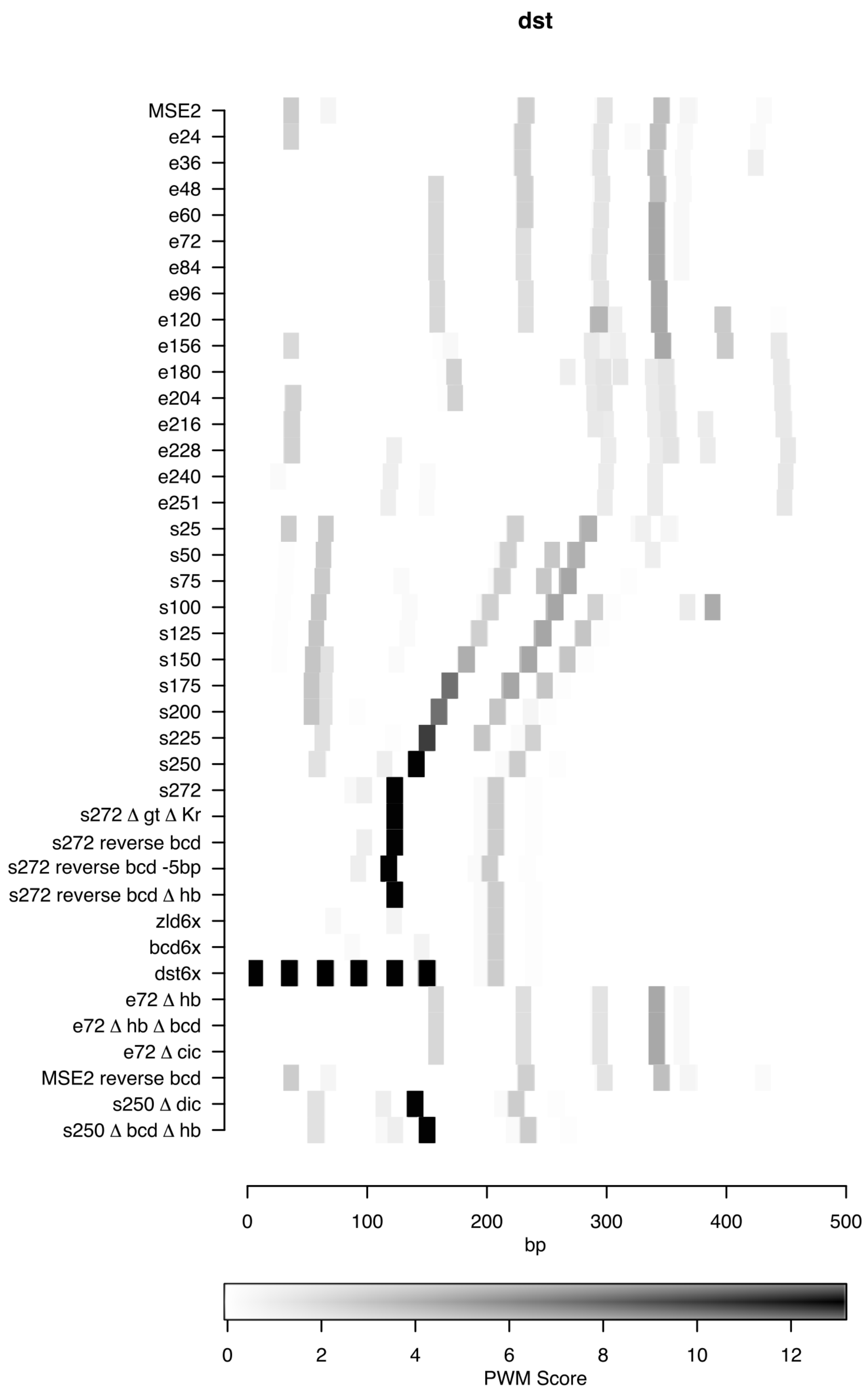


**Figure S9. Binding sites for Dst** For each sequence used in this work, we calculated the Dst PWM score at each position. Motif matches (score>0) are indicated by rectangles that are shaded according to the PWM score, with the legend under the $x$-axis.

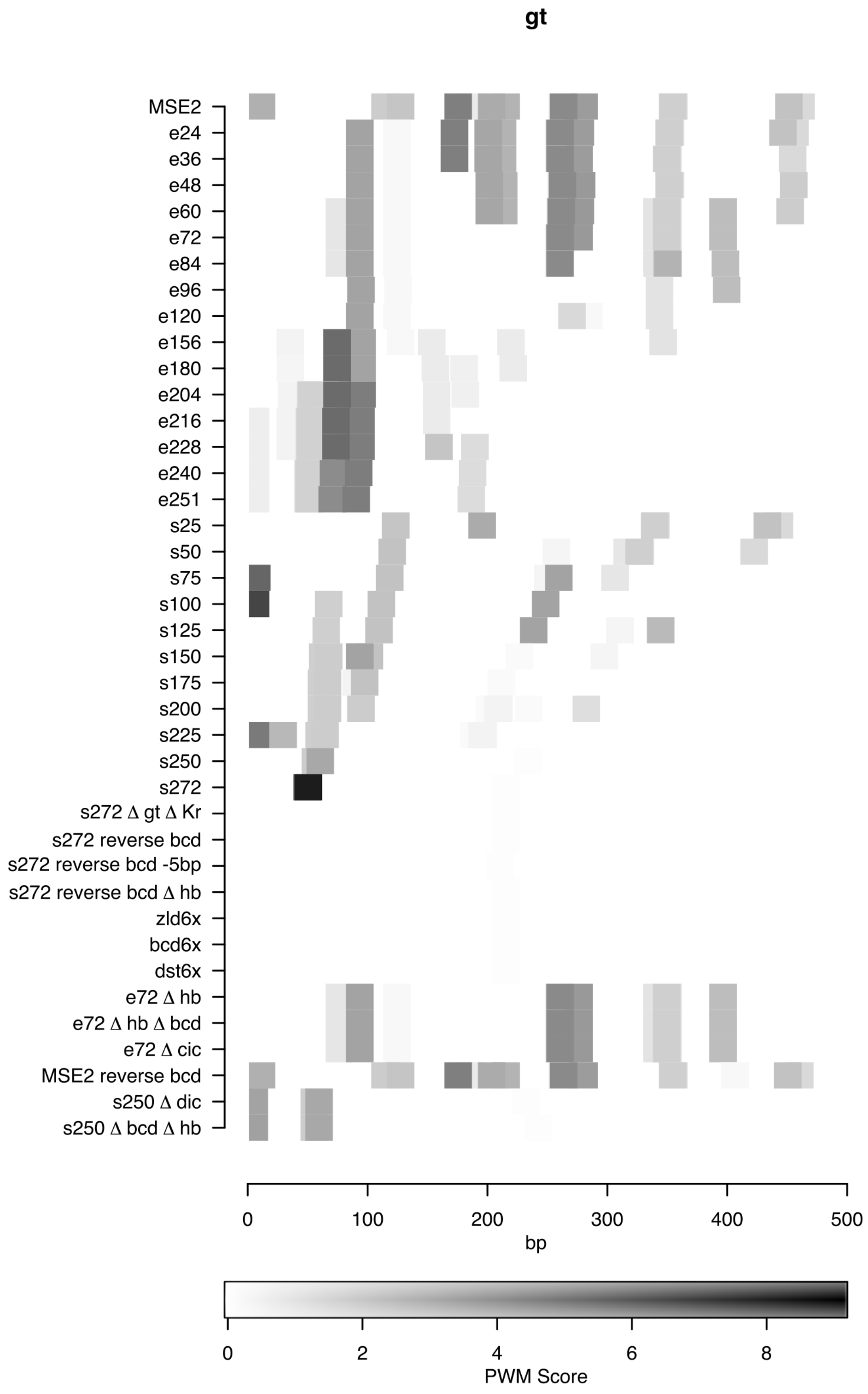


**Figure S10. Binding sites for Gt** For each sequence used in this work, we calculated the Gt PWM score at each position. Motif matches (score>0) are indicated by rectangles that are shaded according to the PWM score, with the legend under the $x$-axis.

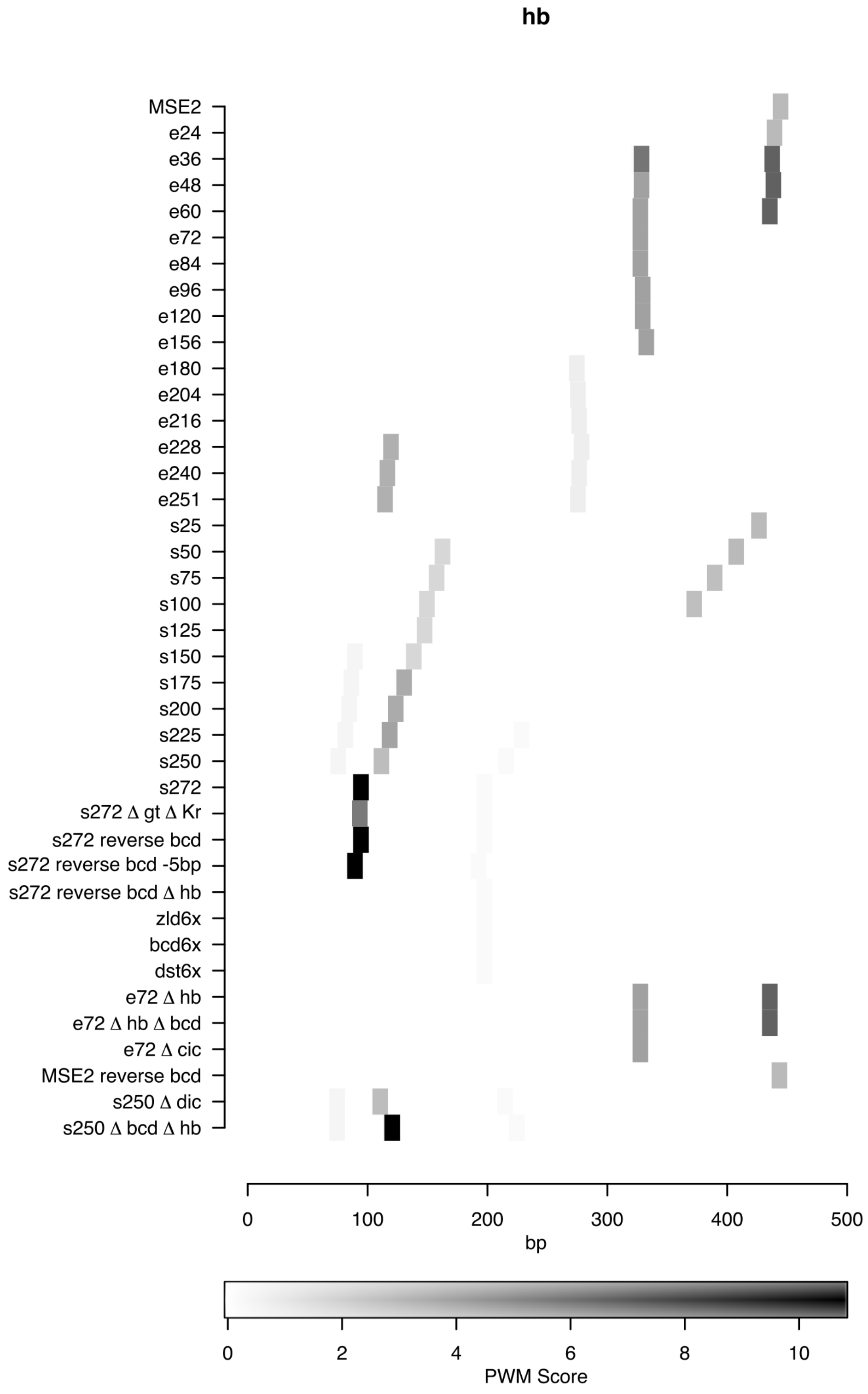


**Figure S11. Binding sites for Hb** For each sequence used in this work, we calculated the Hb PWM score at each position. Motif matches (score>0) are indicated by rectangles that are shaded according to the PWM score, with the legend under the $x$-axis.

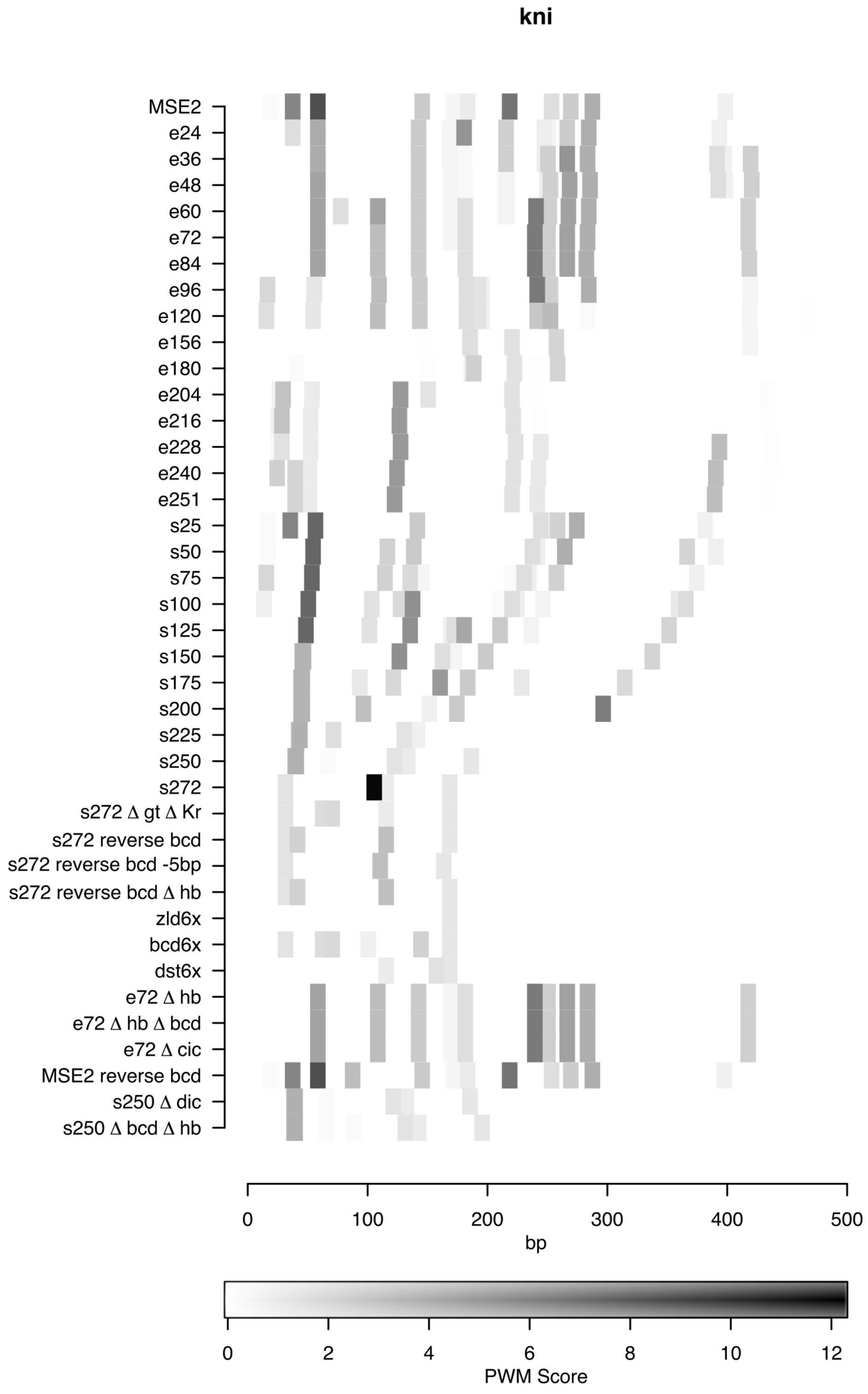


**Figure S12. Binding sites for Kni** For each sequence used in this work, we calculated the Kni PWM score at each position. Motif matches (score>0) are indicated by rectangles that are shaded according to the PWM score, with the legend under the $x$-axis.

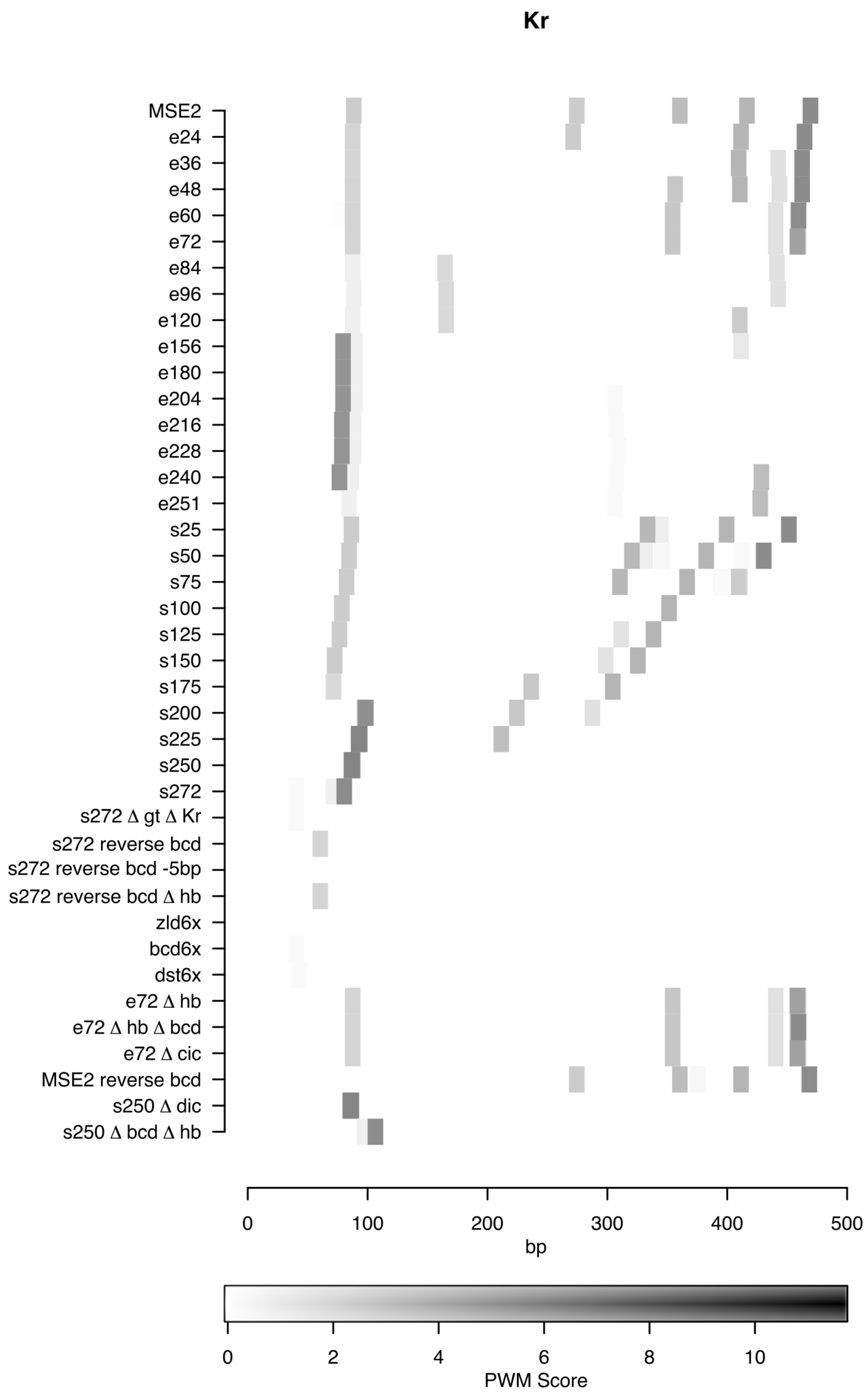


**Figure S13. Binding sites for Kr** For each sequence used in this work, we calculated the Kr PWM score at each position. Motif matches (score>0) are indicated by rectangles that are shaded according to the PWM score, with the legend under the $x$-axis.

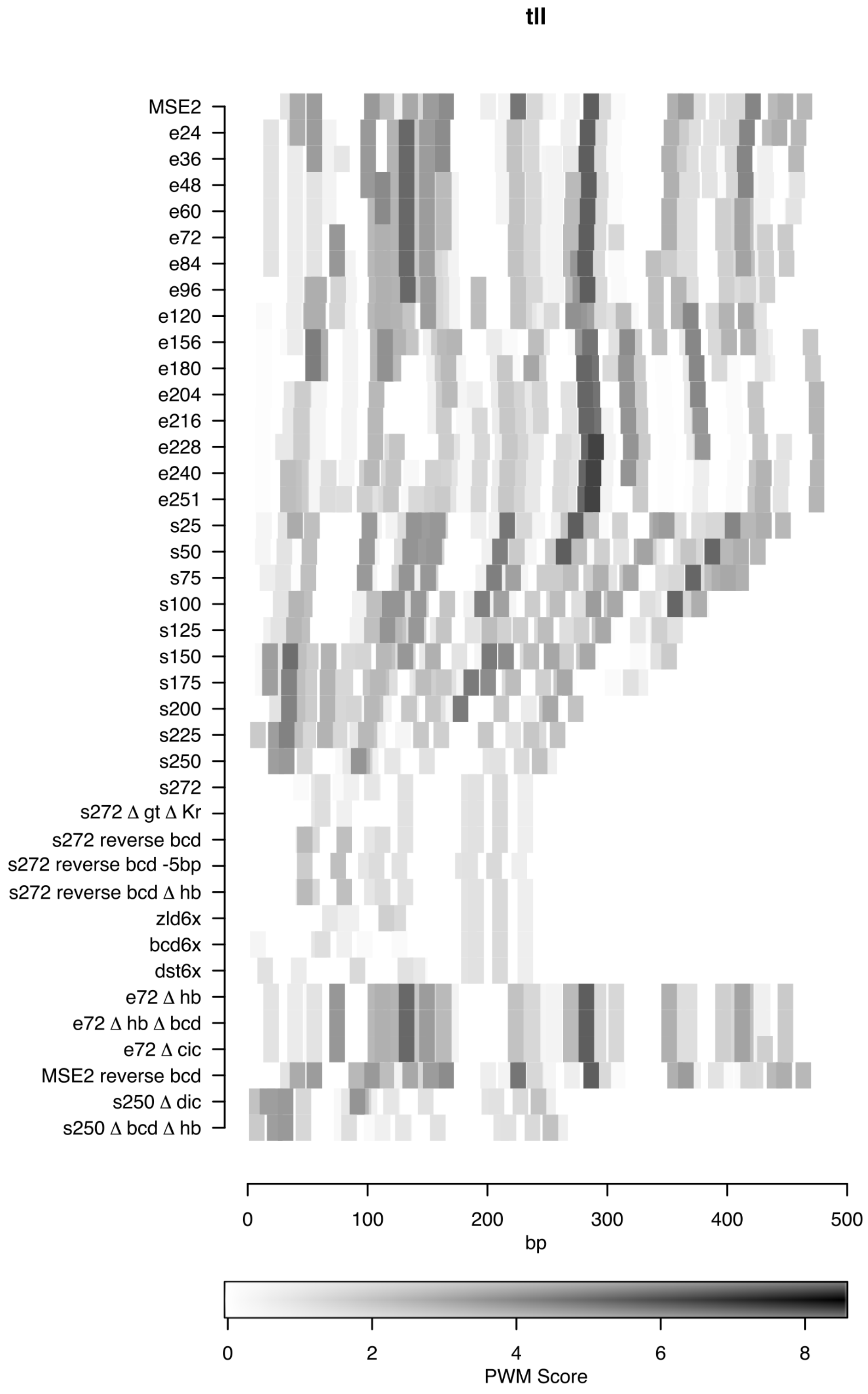


**Figure S14. Binding sites for Tll** For each sequence used in this work, we calculated the Tll PWM score at each position. Motif matches (score>0) are indicated by rectangles that are shaded according to the PWM score, with the legend under the $x$-axis.

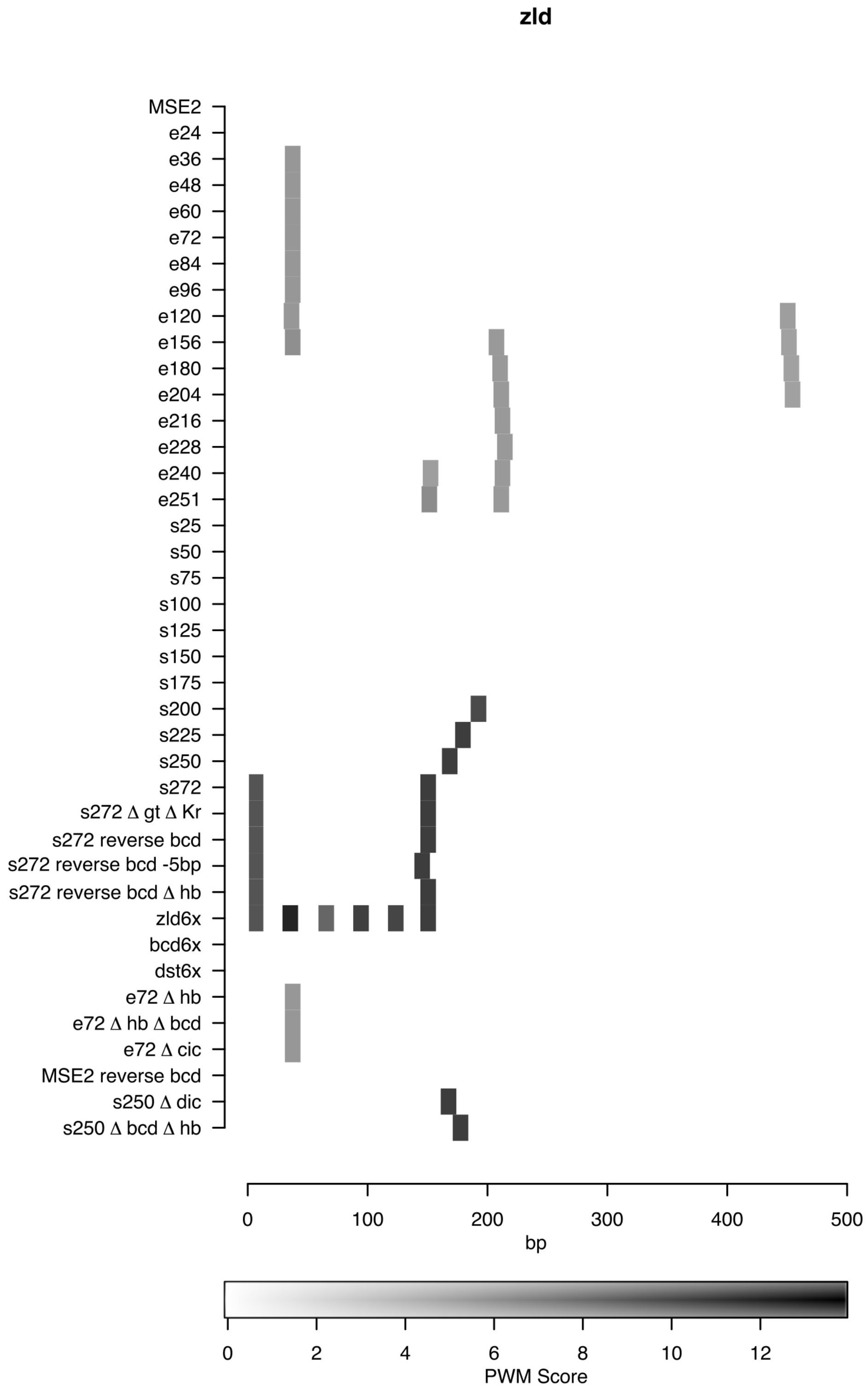


**Figure S15. Binding sites for Zld** For each sequence used in this work, we calculated the Zld PWM score at each position. Motif matches (score>0) are indicated by rectangles that are shaded according to the PWM score, with the legend under the $x$-axis.

| | | Model | | | | | | |
|---|---|---|---|---|---|---|---|---|
| Parameter | TF | 1 | 2 | 3 | 4 | 5 | 6 | 7 |
| $A$ | Bicoid | 0.024022 | 0.037788 | 0.076132 | 0.002134 | 0.089843 | 0.063042 | 0.489863 |
| $\lambda$ | Bicoid | 1.934457 | 4.992031 | 2.162269 | 0.591795 | 0.590000 | 0.590000 | 3.441042 |
| $E^A$ | Bicoid | 2.677410 | 0.062923 | 0.000149 | 16.694600 | 0.000135 | 0.000118 | 0.000108 |
| $E^C$ | Bicoid | 0.999878 | 0.217722 | 0.350294 | 0.683285 | 0.235757 | 0.194850 | 0.139848 |
| $D^C$ | Bicoid | 150.660000 | 158.192000 | 150.630000 | 182.596000 | 186.889000 | 183.882000 | 184.813000 |
| $\omega$ | Bicoid | 499.457000 | 189.210000 | 127.107000 | 34.655300 | 43.989600 | 499.673000 | 9.992570 |
| Threshold | Bicoid | 1.710000 | 1.710000 | 1.710000 | -1.200000 | -1.000000 | -1.000000 | 1.710000 |
| PWM | Bicoid | Bcd_selex | Bcd_selex | Bcd_selex | bicoid_mitomi | bicoid_mitomi | bicoid_mitomi | Bcd_selex |
| $A$ | Caudal | 0.071453 | 0.052367 | 0.026425 | 0.018378 | 0.003403 | 0.008447 | 0.014319 |
| $\lambda$ | Caudal | 4.998159 | 4.976927 | 3.185278 | 9.990996 | 4.999857 | 4.999639 | 4.996867 |
| $E^A$ | Caudal | 0.000103 | 0.000115 | 0.000107 | 0.000136 | 0.000111 | 0.000104 | 0.000153 |
| $E^C$ | Caudal | 0.999899 | 0.337226 | 0.887749 | 0.999925 | 0.999946 | 0.999940 | 0.512709 |
| $D^C$ | Caudal | 16.931600 | 70.875200 | 22.309700 | 11.549400 | 69.292800 | 36.338200 | 69.615400 |
| Threshold | Caudal | 2.501270 | 3.061023 | 2.065076 | 2.992003 | 2.108353 | 2.163674 | 2.728832 |
| $A$ | Stat92E | 3.990583 | 1.099451 | 3.999120 | 3.999022 | 3.999657 | 3.998975 | 3.999516 |
| $\lambda$ | Stat92E | 0.894867 | 2.585787 | 0.696684 | 0.693574 | 0.743904 | 0.735156 | 1.309302 |
| $E^A$ | Stat92E | 0.000193 | 0.000154 | 19.995300 | 19.996900 | 19.999800 | 19.999000 | 0.000392 |
| Threshold | Stat92E | 4.038679 | 2.836359 | 3.631432 | 2.977216 | 3.589654 | 4.098801 | 3.890423 |
| $A$ | Dicheate | 3.953880 | 3.893817 | 0.179983 | 0.051160 | 3.991435 | 0.271187 | 1.163608 |
| $\lambda$ | Dicheate | 1.310409 | 1.983447 | 4.548029 | 2.484454 | 0.929774 | 4.992717 | 2.516001 |
| $E^A$ | Dicheate | 0.000175 | 0.000139 | 0.453824 | 0.000812 | 0.282053 | 0.000156 | 0.000108 |
| Threshold | Dicheate | 4.003251 | 4.794568 | 2.962805 | 3.813067 | 4.909050 | 4.384482 | 2.416283 |
| $A$ | Hunchback | 0.089845 | 2.724241 | 0.041525 | 0.046542 | 0.059710 | 0.054422 | 1.172800 |
| $\lambda$ | Hunchback | 4.735283 | 1.836818 | 4.999943 | 0.591793 | 4.554233 | 4.999490 | 1.839149 |
| $E^Q$ | Hunchback | 0.969302 | 0.999983 | 0.999964 | 0.999967 | 0.999998 | 0.999987 | 0.999985 |
| $E^A$ | Hunchback | 19.546500 | 20.417400 | 29.999000 | 18.683900 | 24.630300 | 27.349300 | 29.979000 |
| $E^D$ | Hunchback | 0.441831 | 0.323916 | 0.581803 | 0.976922 | 0.095546 | 0.000209 | 0.490470 |
| Threshold | Hunchback | 0.630000 | 0.630000 | 0.630000 | -3.250000 | 0.630000 | 0.630000 | 0.630000 |
| PWM | Hunchback | Hb_selex | Hb_selex | Hb_selex | Hb_mitomi | Hb_selex | Hb_selex | Hb_selex |
| $A$ | Zelda | NA | NA | NA | 0.066168 | NA | NA | 0.002172 |
| $\lambda$ | Zelda | NA | NA | NA | 9.999855 | NA | NA | 3.755900 |
| $E^A$ | Zelda | NA | NA | NA | 0.558206 | NA | NA | 19.982600 |
| Threshold | Zelda | NA | NA | NA | 1.487750 | NA | NA | -0.754056 |
| $A$ | Kruppel | 3.997768 | 0.038668 | 2.952886 | 0.865115 | 0.025163 | 0.058992 | 0.055444 |
| $\lambda$ | Kruppel | 0.888617 | 4.040318 | 0.980777 | 1.266060 | 4.997118 | 2.863341 | 4.999282 |
| $E^Q$ | Kruppel | 0.999988 | 0.902770 | 0.999996 | 0.999938 | 0.999990 | 0.999966 | 0.999997 |
| $E^D$ | Kruppel | 0.999654 | 0.999748 | 0.999654 | 0.261343 | 0.414812 | 0.724846 | 0.758756 |
| Threshold | Kruppel | 0.335286 | 2.116363 | 0.072662 | 1.348795 | 0.445604 | 0.536644 | 0.409664 |
| $A$ | Knirps | 0.208940 | 2.233074 | 0.272310 | 0.060819 | 0.267202 | 0.396735 | 0.094753 |
| $\lambda$ | Knirps | 4.998806 | 2.487536 | 1.568829 | 3.879400 | 1.908455 | 1.441430 | 3.153326 |
| $E^Q$ | Knirps | 0.168820 | 0.063714 | 0.999168 | 0.999906 | 0.999376 | 0.999826 | 0.999686 |
| $E^D$ | Knirps | 0.000515 | 0.123624 | 0.999541 | 0.089845 | 0.378947 | 0.034038 | 0.958817 |
| Threshold | Knirps | 4.321854 | 2.234361 | 4.856997 | 5.611598 | 4.105547 | 3.739100 | 2.262279 |
| $A$ | Giant | 0.125367 | 2.530848 | 0.039886 | 0.110539 | 3.996551 | 0.054890 | 3.995412 |
| $\lambda$ | Giant | 4.998344 | 1.713581 | 4.999174 | 2.321564 | 1.273039 | 4.999384 | 1.382071 |
| $E^Q$ | Giant | 0.386106 | 0.723628 | 0.739607 | 0.795631 | 0.492502 | 0.426621 | 0.858064 |
| $E^D$ | Giant | 0.974631 | 0.171351 | 0.999683 | 0.000246 | 0.999547 | 0.999945 | 0.171011 |
| Threshold | Giant | 0.585501 | 0.591202 | 0.504367 | 2.611903 | 0.723453 | 0.646484 | 0.653197 |
| $A$ | Tailless | 0.422458 | 0.023573 | 1.946981 | 0.013529 | 1.014708 | 0.282655 | 0.063704 |
| $\lambda$ | Tailless | 1.304139 | 4.980396 | 0.962922 | 9.761793 | 1.132544 | 1.491165 | 2.710489 |
| $E^Q$ | Tailless | 0.999524 | 0.999485 | 0.999960 | 0.000136 | 0.999794 | 0.998830 | 0.999869 |
| $E^D$ | Tailless | 0.000607 | 0.000255 | 0.000155 | 0.000168 | 0.000241 | 0.000186 | 0.000167 |
| Threshold | Tailless | 1.968527 | 1.977264 | 1.972201 | 2.506890 | 1.812958 | 1.969305 | 1.974659 |
| $\theta$ | NA | 6.147710 | 6.085620 | 5.955810 | 7.478010 | 5.727320 | 5.731930 | 6.242990 |

**Table S1.** Continued on next page...

**Table S1.** Continued from previous page. The model parameters and PWMs that were used generate the sequences in this work are given. All parameters are as described in Kim et. al.[**?** ] and models 1,2, and 3 are discussed in that work where they are called Model 01, Model 06, and Model 07 repectively. Models 5, 6, and 7 were trained as descibed in Kim et. al [**?** ]. Model 4 was trained as described in Martinez et. al [**?** ]. PWMs are specified for factors where the PWM chosen was different among fits.

## 1.2 Position Weight Matrices Used

| > | Bcd_selex | | | | | | | | | | | | | |
|---|---|---|---|---|---|---|---|---|---|---|---|---|---|---|
| A | 83 | 74 | 108 | 48 | 5 | 381 | 379 | 4 | 0 | 5 | 72 | 61 | 65 | 68 |
| C | 114 | 159 | 127 | 149 | 0 | 0 | 0 | 0 | 383 | 340 | 136 | 174 | 166 | 158 |
| G | 106 | 72 | 114 | 11 | 0 | 1 | 4 | 4 | 0 | 2 | 132 | 60 | 52 | 49 |
| T | 80 | 78 | 34 | 175 | 376 | 0 | 0 | 374 | 0 | 34 | 43 | 88 | 100 | 108 |

| > | cad_1hyb | | | | | | | | | |
|---|---|---|---|---|---|---|---|---|---|---|
| A | 9 | 12 | 2 | 3 | 12 | 38 | 0 | 3 | 21 | 1 |
| C | 9 | 6 | 3 | 0 | 0 | 0 | 0 | 0 | 0 | 8 |
| G | 4 | 3 | 3 | 0 | 1 | 0 | 0 | 7 | 14 | 10 |
| T | 11 | 16 | 29 | 34 | 24 | 0 | 38 | 27 | 0 | 1 |

| > | dst_selex | | | | | | | | | | | |
|---|---|---|---|---|---|---|---|---|---|---|---|---|
| A | 0 | 1 | 2 | 1 | 0 | 5 | 3 | 0 | 24 | 28 | 27 | 5 |
| C | 0 | 0 | 1 | 27 | 20 | 16 | 3 | 1 | 3 | 0 | 1 | 7 |
| G | 0 | 1 | 0 | 1 | 6 | 8 | 22 | 27 | 1 | 1 | 0 | 6 |
| T | 28 | 28 | 27 | 1 | 3 | 1 | 2 | 0 | 2 | 1 | 2 | 11 |

| > | dic_1hyb | | | | | | | | | | |
|---|---|---|---|---|---|---|---|---|---|---|---|
| A | 1 | 0 | 0 | 20 | 0 | 0 | 2 | 0 | 1 | 3 | 6 |
| C | 7 | 25 | 17 | 0 | 0 | 0 | 0 | 0 | 2 | 10 | 1 |
| G | 7 | 0 | 0 | 0 | 0 | 3 | 27 | 0 | 4 | 6 | 1 |
| T | 13 | 3 | 1 | 9 | 28 | 26 | 0 | 28 | 22 | 9 | 21 |

| > | hb_selex | | | | | | | | | |
|---|---|---|---|---|---|---|---|---|---|---|
| A | 53 | 1 | 0 | 1 | 0 | 0 | 0 | 280 | 31 | 20 |
| C | 6 | 5 | 1 | 0 | 1 | 2 | 1 | 0 | 43 | 100 |
| G | 224 | 3 | 0 | 0 | 0 | 0 | 0 | 2 | 78 | 109 |
| T | 7 | 279 | 288 | 288 | 288 | 287 | 288 | 5 | 138 | 61 |

| > | Kr_selex | | | | | | | | | |
|---|---|---|---|---|---|---|---|---|---|---|
| A | 17 | 187 | 158 | 0 | 1 | 0 | 8 | 0 | 2 | 44 |
| C | 73 | 5 | 39 | 194 | 194 | 197 | 22 | 1 | 34 | 109 |
| G | 6 | 0 | 0 | 0 | 0 | 0 | 6 | 0 | 1 | 15 |
| T | 101 | 5 | 0 | 2 | 2 | 0 | 161 | 195 | 159 | 29 |

| > | kni_1hyb | | | | | | | | | | | |
|---|---|---|---|---|---|---|---|---|---|---|---|---|
| A | 19 | 25 | 16 | 5 | 0 | 21 | 0 | 17 | 1 | 0 | 25 | 5 |
| C | 0 | 0 | 0 | 9 | 4 | 0 | 0 | 0 | 3 | 26 | 0 | 12 |
| G | 1 | 0 | 0 | 6 | 0 | 5 | 26 | 7 | 18 | 0 | 0 | 7 |
| T | 3 | 0 | 10 | 5 | 21 | 0 | 0 | 1 | 4 | 0 | 0 | 2 |

| > | gt_selex | | | | | | | | |
|---|---|---|---|---|---|---|---|---|---|
| A | 85 | 11 | 776 | 8 | 82 | 0 | 1020 | 1105 | 15 |
| C | 61 | 107 | 24 | 762 | 19 | 555 | 88 | 0 | 378 |
| G | 18 | 359 | 275 | 64 | 996 | 0 | 0 | 0 | 85 |
| T | 941 | 630 | 32 | 274 | 11 | 553 | 0 | 2 | 631 |

> tll_matrix

| | | | | | | | | | |
|---|---|---|---|---|---|---|---|---|---|
| A | 11 | 1 | 1 | 5 | 1 | 11 | 1 | 0 | 0 |
| C | 7 | 2 | 2 | 1 | 2 | 1 | 17 | 2 | 2 |
| G | 0 | 2 | 1 | 0 | 15 | 5 | 0 | 1 | 2 |
| T | 0 | 15 | 16 | 14 | 0 | 3 | 2 | 17 | 15 |

> zld_1hyb

| | | | | | | | | |
|---|---|---|---|---|---|---|---|---|
| A | 54 | 7 | 756 | 0 | 1 | 4 | 756 | 76 |
| C | 0 | 749 | 1 | 0 | 0 | 0 | 5 | 75 |
| G | 478 | 0 | 1 | 731 | 750 | 3 | 0 | 566 |
| T | 229 | 5 | 3 | 30 | 10 | 754 | 0 | 44 |

> Bcd_mitomi

| | | | | | | |
|---|---|---|---|---|---|---|
| A | 0.4446859 | 1.024804 | 0.000000 | 1.308060 | 1.212380 | 0.0000000 |
| C | 0.8241404 | 1.212753 | 0.901402 | 1.386241 | 0.819161 | 1.1815924 |
| G | 0.0000000 | 0.000000 | 1.211622 | 1.455025 | 1.407177 | 1.1908577 |
| T | 0.8472205 | 1.141426 | 1.261173 | 0.000000 | 0.000000 | 0.8257633 |

> Hb_mitomi

| | | | | | | | | |
|---|---|---|---|---|---|---|---|---|
| A | 0.2557999 | 1.8157733 | 2.896855 | 2.954505 | 3.928484 | 3.637427 | 1.759458 | 0.0000000 |
| C | 0.6691087 | 0.8469678 | 2.268623 | 1.556663 | 4.230409 | 2.748917 | 3.135718 | 1.5068606 |
| G | 0.0000000 | 2.5883169 | 2.128065 | 1.824146 | 3.205319 | 3.789288 | 2.927774 | 0.8407924 |
| T | 0.2229569 | 0.0000000 | 0.000000 | 0.000000 | 0.000000 | 0.000000 | 0.000000 | 1.4966308 |

> cic_1hyb

| | | | | | | | | |
|---|---|---|---|---|---|---|---|---|
| A | 1 | 0 | 0 | 18 | 0 | 0 | 0 | 18 |
| C | 10 | 11 | 18 | 0 | 0 | 0 | 10 | 0 |
| G | 0 | 0 | 0 | 0 | 0 | 0 | 8 | 0 |
| T | 6 | 7 | 0 | 0 | 18 | 18 | 0 | 0 |

## 1.3 Sequences

The sequences generated in this work are given below.

>MSE2

CCGGTACTGCATAACAATGGAACCCGAACCGTAACTGGGACAGATCGAAAAGCTGGCCTGGTTTCTCGCTGTGTGTGCCGTGTT
AATCCGTTTGCCATCAGCGAGATTATTAGTCAATTGCAGTTGCAGCGTTTCGCTTTCGTCCTCGTTTCACTTTCGAGTTAGACTTTAT
TGCAGCATCTTGAACAATCGTCGCAGTTTGGTAACACGCTGTGCCATACTTTCATTTAGACGGAATCGAGGGACCCTGGACTATAAT
CGCACAACGAGACCGGGTTGCGAAGTCAGGGCATTCCGCCGATCTAGCCATCGCCATCTTCTGCGGGCGTTTGTTTGTTTGTTTGCT
GGGATTAGCCAAGGGCTTGACTTGGAATCCAATCCCGATCCCTAGCCCGATCCCAATCCCAATCCCAATCCCTTGTCCTTTTCATTAG
AAAGTCATAAAAACACATAATAATGATGTCGAAGGGATTAGGGG

>e24

CCGGTACTGAATACCAGTGGATTCCGAACCGTAACAGGGACAGATCGAAAAGCTGGCCTGGTATCTGCTGTGTGTGCCGTGTT
AATCCGTTGCCACAGCGAGATTGTTAGTCACTTGCAGTTACAGTATTTCGCTGTCGTCCTCGTTTCACTTTCGAGTTAGACTTTATT
GCAGCATCTAGAACAATCGTCGCAGTTCGGTAACACGCTGTGCCATACTATCATTTAGACGGAATCGAGGGACCCTGGACCATAATC
GCACAACGAGACCGGGTTGCGAAGTCAGGGCATTCCGCCGATCTAGCCATCGCCATCTTCTCCGGGTGTTTGTTTGTTTGTTTGCTG
GGATTAGCCAAGGGCTGACTTGGAATCCAATCCCGTCCCTAGCCCGATCCCAATCCCAATCCCAATCCCTTGTCCTTTTCATTAGAAA
GTCATAAAAACACATAATAATGATGTCGAAGGGATTAGGGG

>e36

CCGGTACTGAATACCAGTGGATTCCGAACCGTAACAGGTACAGATCGAAAAGCTGGCCTGGTATCTGCTGTGTGAGCCGTGTTA
ATCCGTTGCCACAGCGAGATTGTTAGTCACTTGCAGTTACAGTATTTCGCTGTCGTCCTCGTTTCACTTTCGAGTTAGACTTTATTGC
AGCATCTATAACAATCGTCGCAGTTCGGTAACACGCTGTGCCATACTATCATTTAGACGGAATCGAGGGACCCTGGACCATAGTCGC
ACAACGAGACCAGTTGCGAAGTCAGGGCATTCCGCCGATCTAGCCATCGCCATCTTCTCCGCGTGTTTTTTGTTTGTTTGCTGGGAT
TAGCCAAGGGCTGACTTGGAATCCAATCCCGTCCCTAGCCCGATCCCGATCCCAATCCCAATCCCTTGTCCTCTTCATTAGAAACTCA
TAAAAACCCATAATAATGATGTCGAAGGGATTAGGGG

>e48

CCGGTACTGAATACCAGTGGATTCCGAACCGTAACAGGTACAGATCGAAAAGCTGGTCTGGTATCTGCTGTGTGAGCCCTGTTA
ATCCGTTGCCACAGCGAGATTGTAAGTCACTTGCAGTTACAGTATTTCGCTGTCGTCCTCGTTTCACTTTCGAGTCTAGACTTTAAT
GCAGCATCTATAACAATCGTCGCAGTTCGGTAACACGCTGTGCCATATCTATCATTTAGACGGAATCGAGGGACCCTGGACCATAGT

CGCACAACGAGACCAATTGCGAAGTCAGGGCATTCCGCCGATCTAGCCATCGCCATCTTCTCCGCGTGTTTTATGATTGTTTGCTGG
GATTAGCCAAGGGTGACTTGGAATCCAATCCCGTCCCTAGCCCGATCCCGATCCCAATCCCAATCCCTTGTCCTCTTCATTTGAAACT
CATAAAAACCCATAATAATGATGCGAAGGGATTAGGGG

>e60

CCGGTACTGAATACCAGTGGATTCCGGACCGTAACAGGTACAGATCGAAAAGCTGGTCTGGTATCTGCTGTGTGACCCCTGTTA
ATCCGTTGCCACAGCGAGAATGTAAGTCACTTGCAGTTACAGTATTTCGCTGTCGTCCTCGTTTCACTTTCGAGTCTAGACTTTAAT
GCAGCATCTATACCAATCGTCGCAGTTCGGTAACACGCTGTGCCATATCTATCATTTAGACGGAATCGAGGGCGCTGGACCATAGTC
GCACAACGAGACCAATTGCGAAGTCAGGGCATTCCGCCGATCTAGCCATCGCCATCTTATCCGCGTGTTTTATGATTGTTTGCGGGA
TTAGCCAAGGGTGACTTGGAATCCAATCCCGTCCATAGCCCGATCCCGACCTAATCCCAATCCCTAGTCCTCTTCATTTGAAACTCAT
AAAAACCCATAATAATGATGCGAAGGGATTAGGGG

>e72

CCGGTACTGAATACCAGTGGATTCCGGACCGTAACAGGTACAGATCGAAAAGCTGGTCTGGTATCTGCTATGTGACCCCTGATA
ATCCGTTGCCACAGCGAGAATGTAAGACACTTGCAGTTACAGTATTTCGCTGTCGTCCTCGTTTCACTTTCGAGTCTAGACTTTAAT
GCAGCATCTATACCAATCGTCGCAGTTCGTTAACACGCTGTCCATATCTATCATTTAGATGGAATCGAGGGCGCTGGACCATAGTCG
CACAACGAGACCAATTGCGAAGTCAGGGCATTCCGCCGATCTAGCCATCGCTCATCTTATCCGCGTGTTTTATGAATGTTTGCGGGA
TTAGCCAAGGGTGACTTGGAATCCAATCCCGTCCATAGCCCGATCCCGACCTAATCCCAATCCCTAGTCCTCTTCATTTGAAACTCAT
ATAAACCCATAATACTGATGGGAAGGGATGAGGGG

>e84

CCGGTACTGAATACCAGTGGATTCCGGACCGTAACAGGTACAGATCGAAAAGCTGGTCTGGTATCTGCTATGTGACCCCTGAGG
ATGCGTTGCCACAGCGAGAATGTAAGACACTTGCAGTTACAGTATTTCGCTGTCGTCCTCGTTTCACTTTCGAGTCTAGCCTTTAAT
GCAGCATCTATACCAATCGTCGCAGTTCGTTAACACGCTGTCCATATCTATCATTTAGATGGAATCGAGGGCGCTGGACCATAGTCG
CACAACGAGACCAATTCGAAGTCAGGGCATTCCGCCGATCTAGCCATCAGCTCATCTTATCCGCGTGTTTTATGAAAGTTTGCGGGA
TTAGCCAATGGTGACTTGGAATCCAATCCCGTCCATAGTTCCCGATCCCGACCTAATCCCAACCCTAGTCCTCTTCATTTGAAACTCA
TATAAACCCATAATACTGATGGGAAGTGATGAGGGG

>e96

CCGGTACTGAATACCAGTGCATTCCGGACCGTAACAGGTACAGATCGAAAAGCTAGGTCTGGTATCTGCTATGCGACCCCTGAG
GATGCGTTGCCACAGCGAGAATGTAAGACACTTGCAGTTACAGTATTTCGCTGTCGTCCTCGTTTCACTTTCGAGTCTAGCCTTTAA
TGCAGCATCTATACCAATCTGTCGCAGTTCGTTAACACGCTGTCCATATCTATCATTTAGATGGAATCGAGGGCGCTGGACCATAGT
CGCACGACGAGACCAATTCGAAGTCAGGGCATTCCGCCGATCTATCCATCAGCTCATCTTATCCGCGTGTTTTATGAAAGTTTGCGG
GATTCGCCAATGGGACTTGGAATCCAATCCCGTCCATAGTTCCCGATCCCGACCTAATCCCAACCCTAGTCATCTTCATTTGAAACTC
ATATAAACCCATAATACCTGATGGGACACTGATGAGGGG

>e120

CGGTACTGAATTCCAGTGCATTCCGGACCGTAACAGGTACAGATCGAAAAGCTAGGTCTGGTATCTGCTATGCGACCCCTGAGG
ATGCGTTGCCACAGCGAGAATGTAAGACACTTGCAGTTACAGTATTTCGACTGTCGTCCTCGTTTCACTTTCGAGTCTAGCCTTTAA
TGCAGCATCTATACCAATCTGTCGCAGTTCGTTAACACGCTGTCCATATCTATCATTTAGATGGAATCGAGCGCGCTGGACCAGAGT
CGCACGACGAGACAATTCGAAGTTAGGGGATTCCGCCGAACATTTCCATCAGCTCACCTTATCCGCGTGTTTTATGAAAGTTTGCGG
GATTCGCCGATGGGACTTGGAATCTAATCCCGTCCATCAGTTCCCGTTCCCGACTAATCCCAACCCAGTCATCTTCATTTGATACTCA
TATATACCCATGATACCTGATGGGACACTCATGAGAGCG

>e156

CAGTACTGAATTCCGAGTGCATTCCGGACCGTTACAGGTACAGCTCGATAAGCTAAGGTCTGGTATCTGCTATGCAACCCCTGA
GGATGCGTTGCCACAGCGAGAATGTAAGACGACTTGCAGTTACAGTATTTCGACTGTCGTCCTCGTTGCACTTTCAAGTCTAGCCTT
GAATGCAGCATCTATACCAATCTGTCGACCCGTACCTTCACACGTTGTCCATATCAATATTCCGATGTGAATCGACCGCGCTGTACCA
GAGTCGCACGACAGAAATTCGAAGTTAAGGGAATCCGCCGAACATTTCCATCAGCTCACTTTATCCGCGTGTTTTATGAAAGTTTGC
GGGATTCGCCGATGGGATTGGAATCTAATCCCGTCCATCAGTTCCCGTTCCCGACTAATCCAACCCAGTAATCTTCATTTGATACTG
ATATATACCCGTGATACCTGATGGGACACTCATGAGACGCG

>e180

CAGTACTGAATTCCGAGTGCATTCTGGACCGTTACAAGTACAGCTCGATAAGCTAAGGTCTGGTATCTGCTATGCAACCCCTGA
GGATGCGTTGCCACAGCGAGAATGTAAGACGACTTTCATGTCTACAGTATTTCGAACGGTCGTCCTCGTTGCACTTTCAAGTCTAGC
CTCGAATGCATGATCTATACCAATCTGTCGACCCGTACCTTCAACGTTGTCCATATCAATATTCCGCTGTGGATGACCGCGCTGTACC
AGAGTCCCACGACAGAAAATCTAAGTTAAGGGAATCCGCCGACACATTTCCATCAGTCTCACTTTATCCGCGTGTTTCATGAAAGTA
TGCGGGATTCGCCGATGGGATTGGAATCTAATCCCGTCCATCAGTTCCCGTTCTCGACTAATCCAACACAGAATCTTCATTTGATAC
TGATATATACCCGTGATACCTGATGGGACACTCATGAGACGCG

>e204

CAGTGCTGAATTCCGAGTGCATTCTGGACCGATTACAAGTAAAGCTGATGATCTAAGGTATGGTATCTGCTATGCAACCCCTGA
GGATGCGTTGCACAGCGAGAATGTAAGACGAGCTTTCATGATCTACAGTATTTCGAACGGGCGTCCTCGTTGCACTTTCAAGTCTAG
CCTCGAATGCATGATCTATCCCAATCTGTCGACCCGTACCTTCAACGTAGTTCATATCAATATGCCGCTGTGGATGACCGCGCTGTA

CCAGAGGCCCACGACAGAAAATCTAAGTTAAGGGAATCCGCCGACACACTTCCATCAGTCTCACTTTATCCGCGTGTTTCATGAAAG
TATGCGGGATTCGCCGATGGGATTGGAATCTAATCCCGTACATCAGTTCCCATTCTCGACTAATCCAACACAGAATCTTAATGTGAT
ACCGATATATACCCGTGATACCTGATGGGACCCTCATGAGACGACG

>e216

CAGTGGTAATTCCGAGTGCATTCTGGACCGATTACAAGTAAAGCTGATGATCTAAGGTATGGTATCTGCTATGCAACCCCTGAG
GATGCGTTGCACAGCGAGAATGTAAGACGAGCTTTCATGATCTACAGTATTTCGAACGGGCGTCCTCAGTTGCACTTTCAAGTCCAG
CCACGAATGTCATGATCAATCCCAATCTGTCGACCCGTACCTTCAACGTAGTTCATATCAATATGCCGCTGTGGATGACCGCGCTGT
ACCAGAGGCCCACGACAGAAAATCTAAGTTAAGGGAATCCTCCGACACACTTCCATCAGTCTCACTTTATCCGCGTGTTTCATGAAA
GTATGCGGGATTCGCGATGGGATTGGAATCTAACTCCCGTACATCAGTTCCCATTCTCGACTAATCCAACACAGAATCTTAAGGTGA
TACCGATATATACCCGTGATACCTGTGGGACCCTCATGAGACGACG

>e228

CAGTGGTAATTCCGAGTGCATTCTGGGCCGATTACAAGTAAAGCTGATGATCTAAGGTATGGTATCTGCTATGCAACCCCTGAG
GATGCGTTGCACAGCGAGAATGTAAGACGAGCTTTCTATGATCTACAGTATTTCGAACGGGCGTTCCTCAGTTGCGCTTTCAAGTCC
AACCACGAATGTCATGAGCAATCCCAATCTGTCGACCCGTACCTTCAACGTAGTTCATATCAATATGCCGCTGTGGATAACCGCGCC
GTGCCAGAGGCCCACGACAGAAAATCTAAGTTAAAGGAATCCTCCGACACACTTCCATCAGTCTCACTTTATCCGCGTGTTTCATGA
AAGTATGCGGGATTCGCGATGGGATTGGAATCTAACTCCCGTACATCAGTTCACCATTCTCGACTAATCCAACACAGAATCTTAAGG
TGATACCGATATATGCCCGTGATACCTGTGGGACCCTCATGAGACGACG

>e240

CAGTGGTAATTCCGAGTGCATTCTGGGCAGATTAAAGTAAAGCTGATGATCTAAGGTATGGTATCTGTATGCAACCCCTGAGGA
TGCGTTGCACAGGAGAATGTAAGACGAGCTTTCTATGATCTACAGTAATTCGAACGGGAGTTCCTCAGTTAGCGCTGTCAAGTCCAA
CCACGAATGTCATGAGCAATCCCAATCTGTCGACCCGTACCTTCAACGTAGTTCATATCAATATGCCGCTGTGGATAACCGCGCCGT
GCCAGAGGCCCACGACAGAAAATCTAAGTTAAAGGAATCCTCCGACACACTTCCATCAGTCTCACTTTATCCGCGTGTTTCATGAAA
GTATGCGGCATTCGCGATGGGATTGGAACTAACACCCGTACATCAGTTCACCATTCTCGACTAATCCAACACAGAATCTTAAGGGTG
ATACCGATATATGCCCGTGATACCTGTGGGACCCTCATGAGACGACG

>e251

CAGTGGTAATTCCGAGTGCATTCTTGGCAGATTAAAGTAAAGCTGATGATCTAAGGTATGGATCTGTATGCAACACCTAGGATG
CGTTGCACAGGAGAATGCAAAACGAGCTTTCTATGATCTACAGTAATTCGAACGGGAGATTCCTCAGTTAGCGGTGTCAAGTCCAAC
CACGAATGTCATGAGCAATCCCAATCTGTCGACCCGTACCTTCAACGTAGTTCATATCAATATGCCGCTGTGGATAACCGCGCCGTG
CCAGAGGCCCACGACAGAAAATCTAAGTTAAAGGAATCCTCCGACACACTTCCAACAGTACTCACTTTATCCGCGTGTTTCATGAAA
GTATGGGCATTCGCGATGGGATTGGAACTAACACCCGTACATCAGTTCACCATTCTCGACTAATCCAACACAGAATCTTAAGGGTGA
TACCGATATATGCCCGTGATACCTGTGGGACCCTCATGAGACGACG

>s25

CCGGTACGCATACAATGGAACCCGAACCGTAACTGGGACAGATCGAAAAGCTGGCCCGGTTTCCCGCTGTGTGTGCCGTGTTAA
TCCGTTTGCCATCAGCGAGATTATTATCAATGCAGTTGCAGCGTTTCGCCTTCGTCCTCGTTTCACTTCGAGTTAGATTTATTCAGCA
TCTTGAAAATCGTCGCAGTTTGGTAACACGCTGTGCCTACTTTCATTTAGACGGAATCGAGGGACCCTGGACTATAATGCACACGAG
ACCGGGTGGAAGTCAGGGCATTCCGCCGAATAGCCATCGCCATCTTCTGCGGCGTTTGTTGTTTGTTTGCAAGGGATTAGCCAAGGC
TTGACTTGGAATCCAATCCCGATCCCAGCCCGATCCCAATCCCAATCCCAATCCCTTGTCCTTTTCATTAGAAGTCATAAAAACACAT
AATAATGATGTCGAAGGGATTAGGGG

>s50

CCGTACGCATACAATGGAACCCGAACCGTAACTGGACAGATCGAAAAGCTGGCCCGGTTTCCCGCTGTGTGTGCCGTGTTAATC
CGTTTGCCATCAGCGAGATTATTATCAATGCATTGCAGCGTTTCGCCTTCGTCCTCGTTTCACTTCGAGTTAGATTTTTCACATCTTG
AAAATCGTCGCAGTTGGTAACACGCTGTGCCTACTTTCGTTTAGACGGAATCAGGGACCCTGGACTATAATGCACACGAGCCGGGTA
AGTCAGGGCATTCCGCCGAATAGCCATCGCCACTTCGCGCGTTTGTTGTTTGATTGCAAGGGATTAGCCAAGGCTTGATTGGAATCC
ATCCCGATCCCAGCCCGATCCGAATCCCAATCAATCCCTTGCCTTTCATTAGAAGTCATAAAAACACATTAATGATGTCGAAGGGATT
AGGG

>s75

CCGTACGCAACAATGGAACGCGAACCGTAACTGGACAGATCGAAAAGCTGGCCCGGTTTCCCGCTGTGTGTGCGTGTTAATCCG
TTTGCCATCAGCGAGATTATTATCAATGCATTGCAGCGTTTCCCTTCGTCCTCATTTACTCGAGTTAGATTTTTCACATCTTGAAAAT
CGTCGCAGTTGGTAACACGCTGTGCCTACTTTCGTTTAGACGGAATCAGACCCTGGACTATAATGCACACGAGCCGGGTAAGTCAGG
CCATTCCGCCGAATGCCTAGCCACTTCGCGCTTTGTTGTTTGATTGCAAGGGATTAGCCGGCTTGATGATCCATCCCGATCCCAGCC
CGATCGAATCCCAATCAATCCCTTGCCTTTCATTAGAAGCTAAAAACACATTAGATGTCGAAGGGATAGGG

>s100

CCTACGCAACATGGAACGCGAACCGTAACTGGACAGATCGAAAGCTGGCCCGGTTTCCCGCTGTTGTGCGTGTTAATCCGTTTG
CCATCAGCGGATTATTATCTGCATTGCAGCGTTCCCTTCGTCCTCATTTGCTCGAGTTAGATTTTTCACATCTGAAAACGTCGCAGTT
GGTAACACGCTGTGCCTACTTTCGTTTAGACGGAATCAGACCCTGGACTATAATGCACACGAGCCGGGTAGTCAGGCCATTCCGCCG

AATGCCTAGCCACTCGCGCTTGTTGTTTGACTGCAAGGAATTAGCCGGCTTGATGATCCATCCCGATCCCAGCCCGATCGAATCCAT
CAATCCCTTGCCTTCATTAGAGCTAAAAACACATAGTGTCGAGGATAGG

>s125

CCTACGCACATGGAACGCGACCGTAACTGGACAGATCGAAAGCTGGCCCGGTTTCCCGCTGTTGTGCGTGTTAATCCGTTTGCC
ATCAGCGGATTATTATCTGCATTGCAGCGTTCCCTTAATCCTCATTTGCTCGAGTTAGATTTTTCACATCTGAAACGTCGCAGTGGT
ACACTGTGCTACTTTGTTTAGACGGAATAGACCCTGGACTAGAATGCACACGAGCCGGGTAGTCAGGCCATTCCGCCGAATGCCTAG
CCACTCGCGCTTGTTGTTTGACCGCAAGGAATTAGCCGGCTTGATGATCATCCCATCCCAGCCGATCGAATCCATCAATCCCTTGCTT
CAGTAGAGCTAAACACATGTCGAGATAGG

>s150

CCTACGCACATGGAAGTGACCGTAACTGGACAGTCGAAGCTGGCCCTATTTCCCGCTGTTGTGCGTGTAATCCGTTTGACAGCG
GCTTATTATCTCATTGCAGCTTCCCTTAATCCTCATTTGCTCGAGTTAGATTTTTCACATCTGAAACGTCGAGTGGTACACTGTGCAC
TTTGTTTAGACGGAAAGACCCTGGACTAGACTGCACACGAGCCAGGTAGTCTGGCCATTCCGCCGAATGCCTAGCCACTCCGCTTGT
TGTTTGACCGCAAGGAATTAGCCGGCTTGATGATCATCCCATCCCAGCCGATCGGATCCATCAATCCCTTGCTCAGTAGAGCTAAAC
ACATCGAATAGG

>s175

CCTACGCACATGGAAGTGACCGTAACTGGACGTCGAAGCTGGCCCTATTTCCCGCTGTTGTGCGTGTAATCCTTGACAGCGGCT
TATTATCCATTGCAGCTCCCTTAATCCTCATTTGCTCGGTTGTTTTTCACATCTGAAACGTCAGTGGACACTGCACTTGTTTGACGGA
AAGACCTGGACTAGACTGCACATGAGCCAGGTAGTCTGGCCATTCCGCCGAATGCCTAGCACCCCTGTTGTTTGACCGCAAGGAATT
AGCCGGCTTGATGATCATCCATCCCAGCCGATCGGATCATCAATCCCTTGCAGTAGAGCTAAACACTCGAATAGG

>s200

CCTACGCACATGGTAGTGACCGTAACTGGACGTCGAAGCTGGCCCTATTTCCCGCTGTTGTGCGTGTAATCCTGCAGCGGCTTA
TTATCCTTGCACCCCTTAATCCTCATTTGCTGGTGTTTTTCACATCTGAAACTCAGTGGACACGCACTTGTTTGACGGAAAGACCTGG
ACTAGACTGCACATGAGCAGGTAGTCTGGCCATTCCGCCAATGCTAGCACCCCTGTTGTTAGACCGCAAGGAATTAGCCGGCTTGAT
ATATCCATCCAGCCGATGGGATACAATCCCTGCAGTAGAGCAAACACTGAATG

>s225

CCTACGCCATGGTAGTGACCGTGACTGACGTCGAAGCTGGCCCTAATTCCCCCTGTTGTGCGTGTAATCCGCAGCGGCTTATTA
TCCTCACCCCTTAATCCTCATTTGCTGGTGTTTTTAATCTAAACTAGGGACACGCACTTGTTTGCCGGAAAGACTGGACTGACTGCA
CTGAGCAGGTAGTCTGGCCATTCCGCCAATGCTAGCACCCCTGTAGTTAGACCCCAAGAAATTAGCCGGTGATATATCCATCCAGCC
GATGGGATACGATCCCTGGTAGAGCAAACACTGAATG

>s250

CCACGACCATGGTAGTGACCGTGACTGACTCGACTGGCCCTAATTCCCCCTGTTGTGCGGTAATCGCAGCGCTTATTATCCTCA
CCCCTTAAACCTCATTTGCTGGTGTTTTTATCAAACTAGGGACACGCACTGTTTCCCGGAAAGACTGACTGACTGACTGAGCAGGTA
GTCTGGCCAGTCCTCCAATGCTAGACCCCTGAGTTAGACCCCAAGAAATTTGCGGTGATATATCATCCAGCCGATGGGATAGATCCC
GGTAGAGAAGCACTGAATG

>s272

ACGCAGGTAGTGACTGACTGACTGACTGGCCCTAATCCCCCCTGTTTGCGTAATCGCAGCCCTAATCCCCCCCTGAAACCCATT
TGCTGGTTTTTTATCAAACTAGGGCACGCACTGTTTCCCGGAAAGACTGACTGACTGACTGAGCAGGTAGTCTGGCCAGTCCTCCAA
TGCTAGACCCCTGAGTTAGACCCCAAGAAATTTGCGGTGATATATCATCGCCGATGGGATAGATCCCGGTAGAGAAGCACTGAATG

>s272 ∆gt ∆Kr

ACGCAGGTAGTGACTGACTGACTGACTGGCCCTAATCCCCCCTGTTCGCGTACTCGCAGCCCTAATGCCCCCCTGAATACCAAT
TGATGGTTTTTAGTCTAACCGTATAACGCACTGTTTCCCGGAAAGACTGACTGACTGACTGAGCAGGTAGTCTGGCCAGTCCTCCAA
TGCTAGACCCCTGAGTTAGACCCCAAGAAATTTGCGGTGATATATCATCGCCGATGGGATAGATCCCGGTAGAGAAGCACTGAATG

>s272 reverse bcd

ACGCAGGTAGTGACTGACTGACTGACTGGCCCTAATCCCCCCAGTTTGCTTACTCGCAGGGGGGGATTAGGGCCTGAAACCGAGT
TGCTGGTTTTTTATCAAACTACGAAGCGCACTGTTTCCCGGAAAGACTGACTGACTGACTGAGCAGGTAGTCTGGCCAGTCCTCCAA
TGCTAGACCCCTGAGTTAGACCCCAAGAAATTTGCGGTGATATATCATCGCCGATGGGATAGATCCCGGTAGAGAAGCACTGAATG

>s272 reverse bcd -5bp

ACGCAGGTAGTGACTGACTGACTGACTGGCCCTAATCCCCCCAGTTGTTCTGCGGGGGGATTAGGGCCTGAAACCGAGTTGCT
GGTTTTTTATCAAACTACGAAGCGCACTGTTTCCCGGAAAGACTGACTGACTGACTGAGCAGGTAGTCTGGCCAGTCCTCCAATGCT
AGACCCCTGAGTTAGACCCCAAGAAATTTGCGGTGATATATCATCGCCGATGGGATAGATCCCGGTAGAGAAGCACTGAATG

>s272 reverse bcd ∆hb

ACGCAGGTAGTGACTGACTGACTGACTGGCCCTAATCCCCCCAGTTTGCTTACTCGCAGGGGGGGATTAGGGCCTGAAACCGAGT
TGCTGGTTCTGTATCAAACTACGAAGCGCACTGTTTCCCGGAAAGACTGACTGACTGACTGAGCAGGTAGTCTGGCCAGTCCTCCAA
TGCTAGACCCCTGAGTTAGACCCCAAGAAATTTGCGGTGATATATCATCGCCGATGGGATAGATCCCGGTAGAGAAGCACTGAATG

>zld6x

ACGCAGGTAGTGACTGACTGACTGACTGGCCGCAGGTAGCCCTGTTCGCGTACTCGCTGCGGCATGTAGCCCCTGAATACCAAT TGATCGGCAGGTAGCTAACCGTATAACGCACTCTTGCAGGTAGAGACTGACTGACTGACTGAGCAGGTAGTCTGGCCAGTCCTCCAA TGCTAGACCCCTGAGTTAGACCCCAAGAAATTTGCGGTGATATATCATCGCCGATGGGATAGATCCCGGTAGAGAAGCACTGAATG

>bcd6x

CCGCTAAGCCCCCCGGACTGACTGACTGGCCCTAATCCCCCCTGTTCGCGTACTCGCAGCCCTAATGCCCCCCTGAATACCAAT TCACCATAATCCGCCCCACCGTATAGGGAACCCCCGAATCCGCCCCCGGACTGACTGACCCACTAATCCGCCCGGCCAGTCCTCCAAT GCTAGACCCCTGAGTTAGACCCCAAGAAATTTGCGGTGATATATCATCGCCGATGGGATAGATCCCGGTAGAGAAGCACTGAATG

>dst6x

TTTCCCGGAAAGACTGACTGACTGACTGGTTTCCCGGAAACCTGTTCGCGTACTCGCAGTTTCCCGGAAACCCTGAATACCGAT TGATTTCCCGGAAACTAACCGTATAACGCACTGTTTCCCGGAAAGACTGACTGACTGACTTTTCCCGGAAACTGGCCAGTCCTCCAA TGCTAGACCCCTGAGTTAGACCCCAAGAAATTTGCGGTGATATATCATCGCCGATGGGATAGATCCCGGTAGAGAAGCACTGAATG

>e72 Δhb

CCGGTACTGAATACCAGTGGATTCCGGACCGTAACAGGTACAGATCGAAAAGCTGGTCTGGTATCTGCTATGTGACCCCTGATA ATCCGTTGCCACAGCGAGAATGTAAGACACTTGCAGTTACAGTATTTCGCTGTCGTCCTCGTTTCACTTTCGAGTCTAGACTTTAAT GCAGCATCTATACCAATCGTCGCAGTTCGTTAACACGCTGTCCATATCTATCATTTAGATGGAATCGAGGGCGCTGGACCATAGTCG CACAACGAGACCAATTGCGAAGTCAGGGCATTCCGCCGATCTAGCCATCGCTCATCTTATCCGCGTGTTTTATGAATGTTTGCGGGA TTAGCCAAGGGTGACTTGGAATCCAATCCCGTCCATAGCCCGATCCCGACCTAATCCCAATCCCTAGTCCTCTTCATTTGAAACTCAT AAAAACCCATAATACTGATGGGAAGGGATGAGGGG

>e72 Δhb Δbcd

CCGGTACTGAATACCAGTGGATTCCGGACCGTAACAGGTACAGATCGAAAAGCTGGTCTGGTATCTGCTATGTGACCCCTGATA ATCCGTTGCCACAGCGAGAATGTAAGACACTTGCAGTTACAGTATTTCGCTGTCGTCCTCGTTTCACTTTCGAGTCTAGACTTTAAT GCAGCATCTATACCAATCGTCGCAGTTCGTTAACACGCTGTCCATATCTATCATTTAGATGGAATCGAGGGCGCTGGACCATAGTCG CACAACGAGACCAATTGCGAAGTCAGGGCATTCCGCCGATCTAGCCATCGCTCATCTTATCCGCGTGTTTTATGAATGTTTGCGGGA TTAGCCAAGGGTGACTTGGAATCCAATCCCGTCCATAGCCCGATCCCGACCTAATCCCAATCCCTAGTCCTCTTCATTTGAAACTCAT AAAAACCCATAATACTGATGGGAAGGGATTAGGGG

>e72 Δcic

CCGGTACTGAATACCAGTGGATTCCGGACCGTAACAGGTACAGATCGAAAAGCTGGTCTGGTATCTGCTATGTGACCCCTGATA ATCCGTTGCCACAGCGAGAATGTAAGACACTTGCAGTTACAGTATTTCGCTGTCGTCCTCGTTTCACTTTCGAGTCTAGACTTTAAT GCAGCATCTATACCAATCGTCGCAGTTCGTTAACACGCTGTCCATATCTATCATTTAGATGGAATCGAGGGCGCTGGACCATAGTCG CACAACGAGACCAATTGCGAAGTCAGGGCATTCCGCCGATCTAGCCATCGCTCATCTTATCCGCGTGTTTTATGATTGTTTGCGGGA TTAGCCAAGGGTGACTTGGAATCCAATCCCGTCCATAGCCCGATCCCGACCTAATCCCAATCCCTAGTCCTCTTCATTTGAAACTCAT ATAAACCCATAATACTGATGGGAAGGGATGAGGGG

>MSE2 reversed bcd

CCGGTACTGCATAACAATGGAACCCGAACCGTAACTGGGACAGATCGAAAAGCTGGCCTGGTTTCTCGCTGTGTGTGCCGTGTg gattaGTTTGCCATCAGCGAGATTATTAGTCAATTGCAGTTGCAGCGTTTCGCTTTCGTCCTCGTTTCACTTTCGAGTTAGACTTTATT GCAGCATCTTGAACAATCGTCGCAGTTTGGTAACACGCTGTGCCATACTTTCATTTAGACGGAATCGAGGGACCCTGGACTATAATC GCACAACGAGACCGGGTTGCGAAGTCAGGGCATTCCGCCGATCTAGCCATCGCCATCTTCTGCGGGCGTTTGTTTGTTTGTTTGCTG GGATTAGCCAAGGGCTTGACTTGGAATCCATCCCGATCCCTAGCCCGATCCCAATCCCAcaagggattgggatTCCTTTTCATTAGAAAGTC ATAAAAACACATAATAATGATGTCGAAGGGATTAGGGG

>s250 Δdic

CCACGCCACGGAAGTGACCGTGACTGACTCGACTGGCCCTAATTCCCCCTGTTGTGCGGTAATCGCAGCGCTTATTATCCTCAC CCCTTAAACCTCATTTGCTGGTGTTTTTATCAAACTAGGGACACGCACTGTTTCCCGGAAAGACTGACTGACTGACTGAGCAGGTAG TCTGGCCAGTCCTCCAATGCTAGACCCCTGAGTTAGACCCCAAGAAATTTGCGGTGATATATCATCCAGCCGATGGGATAGATCCCG GTAGAGAAGCACTGAATG

>s250 Δbcd Δhb

CCACGCCATGGTAGTGACCGTGACTGACTCGACTGGCCCTAATTCCCCCTGTTGTGCGGTAATCGCAGCGCTTATTATCCTCAC GCCCTAATCCCCCCCTGAAACCCATTTGCTGGTTTTTTATCAAACTAGGGACACGCACTGTTTCCCGGAAAGACTGACTGACTGACT GAGCAGGTAGTCTGGCCAGTCCTCCAATGCTAGACCCCTGAGTTAGACCCCAAGAAATTTGCGGTGATATATCATCCAGCCGATGGG ATAGATCCCGGTAGAGAAGCACTGAATG

>5' Extention Primer

TGGGTTTTATTAACTTACATACATACTAGAATTCGAGCTCGCCCGGGGATC

>3' Extention Primer

GTTGTTGACTGTGCGGCGGTCACAGCTCGAGTGTGCTGCTCTCAGCCACCCCGCGCCCTTTTATACCGCTGCGCTC
